# Depth Profiling of Oxygen Migration in Ta/HfO_2_ Stacks during Ionic Liquid Gating

**DOI:** 10.1021/acsami.5c22179

**Published:** 2026-01-13

**Authors:** Beatrice Bednarz, Martin Wortmann, Olga Kuschel, Fabian Kammerbauer, Mathias Kläui, Andreas Hütten, Joachim Wollschläger, Gerhard Jakob, Timo Kuschel

**Affiliations:** † Institute of Physics, 9182 Johannes Gutenberg University Mainz, Staudingerweg 7, 55128 Mainz, Germany; ‡ Faculty of Physics, 9167 Bielefeld University, Universitätsstraße 25, 33615 Bielefeld, Germany; § Faculty of Physics, 9186 Osnabrück University, Barbarastraße 7, 49076 Osnabrück, Germany

**Keywords:** voltage control of magnetism, ionic liquid, oxygen doping, X-ray photoelectron spectroscopy, X-ray reflectivity, depth profile

## Abstract

Ionic liquid (IL) gating has emerged as a powerful tool to control the structural, electronic, optical, and magnetic properties of materials by driving ion motion at solid interfaces. In magneto-ionic systems, electric fields are used to move ions, typically oxygen, from a donor layer into an underlying magnetic metal. Although oxygen distribution is key to enabling precise and stable control in magneto-ionic systems, the spatial distribution and voltage-dependence of oxygen incorporation in such nanoscale stacks remain unknown. Here, we quantify oxygen depth profiles and oxide formation in Si/SiO_2_/Ta­(15)/HfO_2_(*t*) films after IL gating as a function of the gate voltage and HfO_2_ capping thickness (*t* = 2 and 3 nm). X-ray reflectivity and X-ray photoelectron spectroscopy measurements revealed a threshold electric field of ≈−2.8 MV/cm to initiate oxygen migration from HfO_2_ into metallic Ta. The resulting Ta_2_O_5_ thickness increases linearly with gate voltage, reaching up to 4 nm at −3 V gating. Notably, the required electric field rises with oxide thickness, indicating a progressively growing barrier for thicker oxide films. The Ta/Ta_2_O_5_ interface remains atomically sharp for all gate voltages. This suggests that complete Ta_2_O_5_ layers form sequentially before further oxygen penetration, with no sign of deeper diffusion into bulk Ta. Thinner capping layers enhance oxidation, relevant for optimized stack design. Additionally, indium migration from the indium tin oxide electrode to the sample surface was observed, which should be considered for surface-sensitive applications. These insights advance design principles for magneto-ionic and nanoionic devices requiring precise interface engineering.

## Graphical abstract

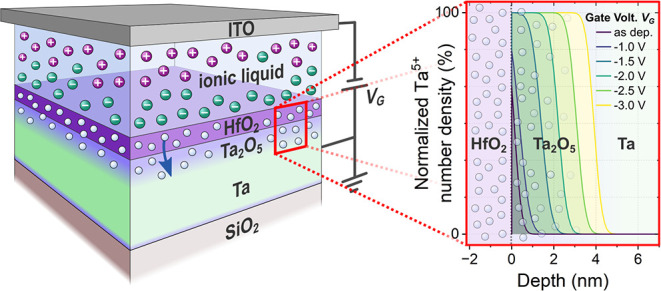

## Introduction

Ionic liquids (IL) are well-known for their unique combination of high electrochemical capacitance, usually low toxicity, negligible volatility, and broad thermal stability range.^1^ Their use in ionic gating enables the application of extremely large electric fields (10–100 MV/cm),^2^ far exceeding the dielectric breakdown limits of conventional SiO_2_-based metal–oxide–semiconductor field-effect transistors (MOSFETs).^2^ These strong electric fields allow for field-driven ion motion, enabling reversible tuning of electronic, magnetic, optical, and structural properties.^2–6^ In this regard, IL gating complements solid-state gating,^7–9^ which typically requires comparatively thick dielectric layers to ensure electric insulation. The electric double layer of an IL is only about 1 nm thick, which enables the large electric fields characteristic of IL gating.^2^ Similarly to solid-state gating, IL gating has proven effective in controlling a wide range of magnetic interactions, including magnetic anisotropy,^10,11^ exchange bias,^12^ Dzyaloshinskii–Moriya interaction,^13^ and Ruderman–Kittel–Kasuya–Yosida coupling.^14^ This expanding toolbox of electric-field control over magnetic states opens possible pathways to ultralow-power data storage, neuromorphic hardware, and spin-based sensors.^10,15–19^

Among various mobile species, oxygen ions are particularly attractive for magneto-ionic control due to their reactivity with a broad range of materials and the availability of multiple oxygen donor layers. Despite the widespread use of voltage-induced oxygen migration in functional magnetic stacks, the spatial profile of oxygen incorporation remains unknown. This is critical because the penetration depth, concentration profile, and sharpness of the resulting oxide/metal interface influence bulk magnetic and electronic properties through changes in oxidation state or chemical composition.^10^ The oxygen depth profile might also affect adjacent functional layers in ultrathin or multilayer architectures. For instance, in exchange bias systems or synthetic antiferromagnets, magnetic properties are set at buried interfaces. Information on the oxygen penetration depth is essential for determining the optimal thickness of layers above the critical interface to either modulate or protect the interface. Additionally, recent work has shown that introducing a thin tantalum (Ta) insertion layer below the IL can significantly enhance the cyclability of oxide-based magneto-ionic devices,^20^ suggesting that the oxygen incorporation profile in such layers may play a key role in device stability and performance. However, it is neither known which amount of oxygen ions penetrates far down into the material and how the oxygen profile looks after IL gating, nor which process underlies the migration. A more detailed understanding of the oxygen distribution is therefore key to enabling precise and stable control in magneto-ionic systems.

In this study, we combine X-ray reflectivity (XRR) with angle- and energy-resolved X-ray photoelectron spectroscopy (XPS) to study the oxygen depth distribution in Ta as a function of the IL gate voltage in two systems with different hafnium oxide (HfO_2_) capping layer thicknesses (see Figure 1a). Ta was chosen as the investigated metal due to its very smooth growth, making it an ideal model system for the investigation of homogeneous metallic films. Because of this very smooth growth and its ductility and corrosion resistance, Ta is both a common seed layer as well as an important coating in a variety of applications.^21,22^ The Ta thickness was set to 15 nm to significantly exceed the XPS information depth, as approximately 95% of the XPS signal originates from the upper 4.5 nm of the Ta layer (three times the effective attenuation length (EAL), see SI 4). This ensures a clean signal without any influence of the substrate layer. HfO_2_ was selected as a capping layer due to its high-κ dielectric properties and high oxygen ion mobility, making it a common component in IL gating stacks.^23^ Oxygen ions were driven into the Ta layer by applying gate voltages between −1.0 and −3.0 V using IL gating.

**Figure 1:**
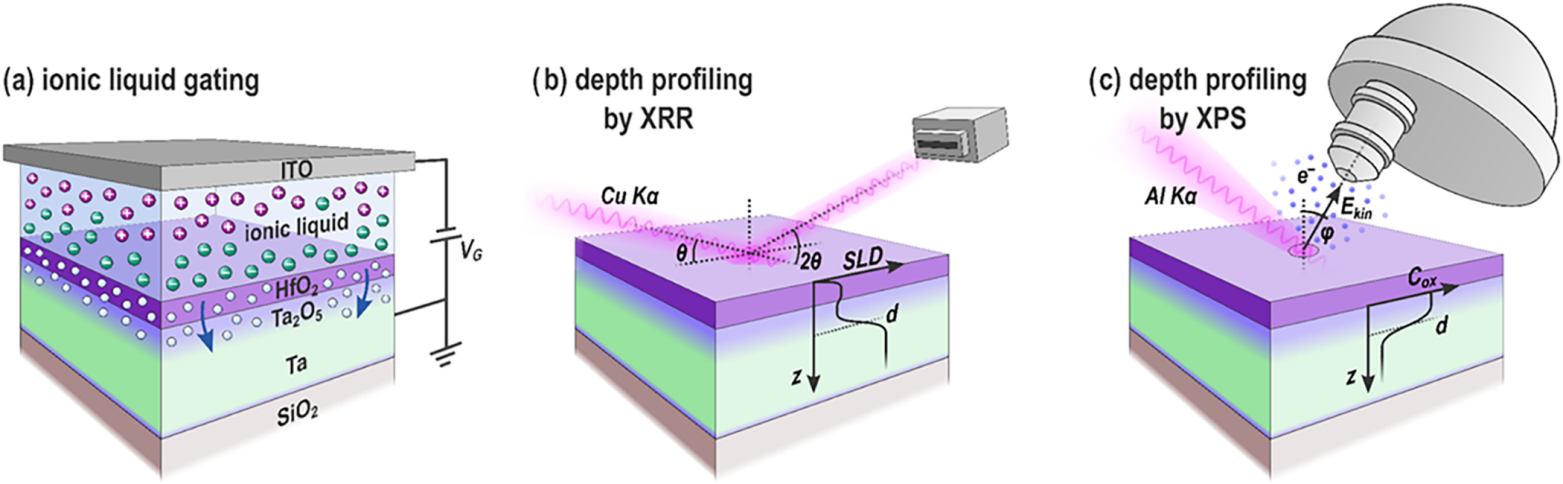
(a) Schematic of the investigated material stack during IL gating. By applying a negative gate voltage (*V*_G_) the IL is polarized and transmits the gate voltage to the sample. There, oxygen ions (white spheres) are pushed from the HfO_2_ into the Ta layer, forming a Ta_2_O_5_ layer at the interface. (b) Sketch of the XRR setup. By shining X-rays at grazing angles onto the sample and measuring their reflection in a specular geometry, information on the average layer thickness and interface width of the different layers can be obtained. The quantity which defines the scattering is the scattering length density (SLD, see SI 8 for more details). (c) Sketch of the XPS setup, in which the photoelectrons are detected as a function of their emission angle φ. From their kinetic energy *E*_kin_, the binding energy of the respective element can be obtained, which provides information about the element and its oxidation state. By varying the investigated emission angle, the depth distribution from which the photoelectrons stem changes. Using this, a depth profile of the concentration of oxidized Ta atoms (*C*_ox_) can be deduced.

We demonstrate that the average oxide layer thickness increases linearly with gate voltage when voltages above −1.0 V are applied. For thinner HfO_2_ capping layers, the average oxide thickness at the same applied gate voltage is larger. The oxide/metal interface thereby remains atomically sharp, only a few Å wide, at all gate voltages. Notably, indium ions from the indium tin oxide (ITO) electrode above the IL migrate to the sample surface during IL gating. This is important to consider when designing surface-sensitive applications.

## Results and Discussion

Oxygen migration in magneto-ionic systems is investigated by XRR and XPS. As a model system, we studied Si/SiO_2_/Ta­(15)/HfO_2_(*t*) heterostructures. Here, the number in the parentheses denotes layer thickness in nm and *t* = 2 or 3 nm (Figure 1a). To resolve the oxygen distribution within the Ta layer, we first carried out XRR measurements (Figure 1b). The results for the samples with a 2 nm HfO_2_ capping layer are shown in Figure 2. The XRR fits and corresponding scattering length density (SLD) graphs are exemplarily shown for the as-deposited state (Figure 2a) and after 10 min of maximum gate voltage of −3 V (Figure 2b). All other fits can be found in the Supporting Information (SI 2). For the fit model, we considered the HfO_2_ capping layer, an oxidized Ta_2_O_5_ layer, which is the only stable oxide of Ta,^24^ the metallic Ta layer, a thin bottom Ta_2_O_5_ layer between the Ta and the SiO_2_ resulting from oxygen scavenging, as well as the SiO_2_ substrate.

**Figure 2:**
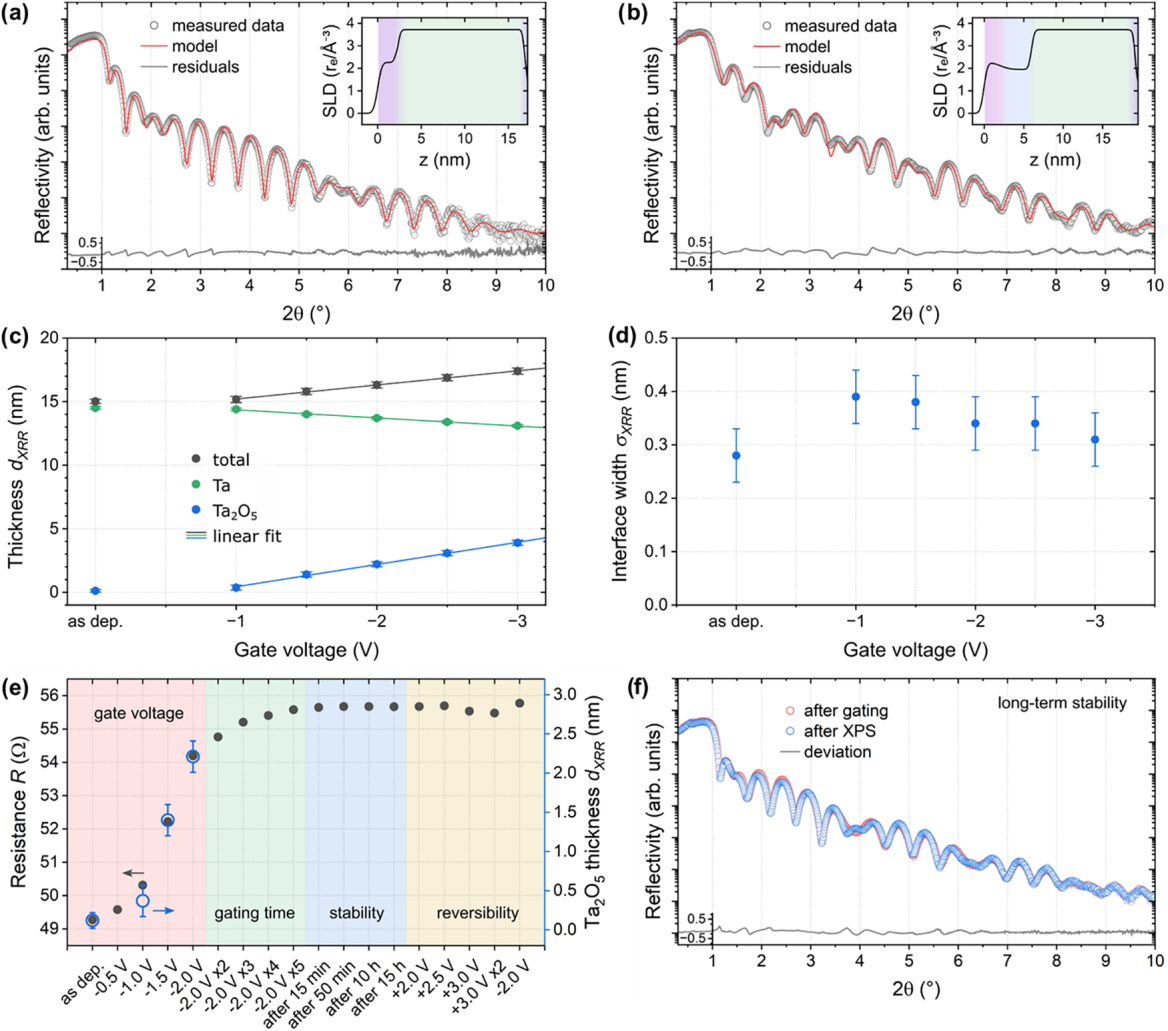
XRR analysis of the sample Si/SiO_2_/Ta­(15)/HfO_2_(2) as a function of the applied gate voltage. (a, b) XRR fits for the samples in the as-deposited state and after applying −3.0 V for 10 min. The fit residuals are calculated as log­(model)–log­(data) and plotted on a linear scale below the graphs. The insets show the corresponding SLD profiles (starting from air with SLD = 0, to HfO_2_ marked in purple, Ta_2_O_5_ in blue, Ta in green and the bottom Ta_2_O_5_ due to the interface with SiO_2_ in blue again). (c) Evolution of the thicknesses of Ta, Ta_2_O_5_ and the total thickness (sum of Ta, Ta_2_O_5_ and the Ta_2_O_5_ below the Ta) as a function of the gate voltage, applied for 10 min each. (d) Interface width σ at the upper Ta_2_O_5_/Ta-interface. The XRR fits and SLD profiles at the other gate voltages, as well as details on the fits and all estimated uncertainties can be found in SI 1 and 2. (e) Influence of gate voltage and gating time, as well as stability and reversibility of the gating-induced oxidation. All measurements were performed consecutively on the same sample by applying the gate voltages for 10 min each and measuring the longitudinal resistance right after turning off the voltage, without removing the IL or top electrode. For comparison, the Ta_2_O_5_ thicknesses determined by XRR are shown as blue open circles for the gate voltages at which XRR measurements were performed. (f) XRR measurement performed right after gating at −1.5 V for 10 min compared to a measurement recorded after XPS (two months later).

The SLD profiles of the samples depict a clear and only a few Å wide interface between the Ta and the upper, gating-induced Ta_2_O_5_. The two oxide layers above the Ta (Ta_2_O_5_ and HfO_2_) cannot be clearly distinguished, as can be seen from the very broad SLD transition between the two in the inset of Figure 2b. The reason is the very similar density and therefore similar SLD of Ta_2_O_5_ (ρ = 8.2 g/cm^3^ ≙ SLD = 1.95 *r*_e_/Å^3^) and HfO_2_ (ρ = 9.7 g/cm^3^ ≙ SLD = 2.27 *r*_e_/Å^3^). For the same reason, additional intermediate TaO_*x*_ compositions cannot be excluded if their densities lie close enough to the densities of Ta_2_O_5_ and HfO_2_. However, the XPS spectra reveal no spectroscopically resolvable evidence for additional intermediate oxides, and a model including only the dominant oxidation states already provides an adequate description of the data as shown further below. While the presence of minor amounts of intermediate Ta oxides below the detection limit cannot be completely excluded, they would be energetically less favorable than Ta_2_O_5_.^24^

To disentangle the Ta_2_O_5_ and HfO_2_ layer thicknesses, the HfO_2_ thickness was fixed to its value in the as-deposited state for all further gating steps. This is a reasonable assumption as the HfO_2_ layer shows no sign of an induced metallic phase after gating, as demonstrated by XPS (Figure 3). The possible error on the Ta_2_O_5_ thickness, which is induced by this assumption, is included in its uncertainty (see SI 1 for more information on the estimated uncertainties). Similarly, the thickness and interface width of the bottom Ta_2_O_5_ were fixed to the value obtained from the as-deposited state, assuming that the applied electric field is screened by the Ta layer and does not influence the bottom Ta_2_O_5_ layer. In the following, Ta_2_O_5_ will therefore always refer to the upper Ta_2_O_5_ layer induced by the IL gating.

**Figure 3:**
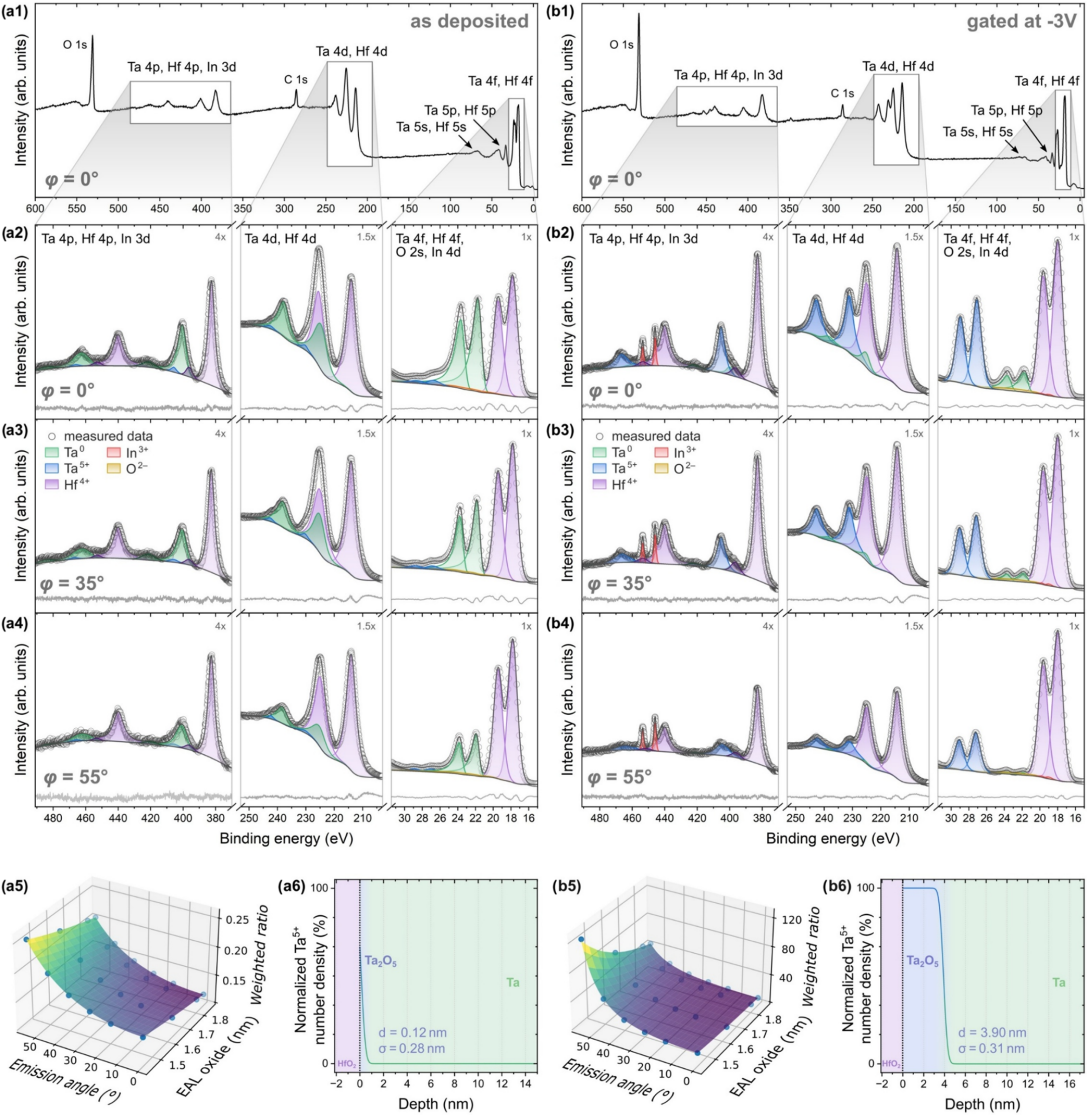
XPS spectra for the sample with 2 nm HfO_2_ (a) in the as-deposited state and (b) after the maximum applied gate voltage of −3.0 V. (a1, b1) Survey spectra and (a2–4, b2–4) fitted core-level spectra for the peak regions Ta 4p, 4d, and 4f for three different emission angles (0, 35, and 55° respective to the surface normal, for which cos(35°) is approximately in the middle between the cosines of the other two angles). The fitted core-level spectra of the Ta 5s and O 1s regions, as well as the other angles and gating steps, can be found in SI 7. The Ta 5p region (starting at 31 eV toward higher binding energies) was not fitted because of the high number of peaks with large overlap and the resulting high uncertainty of the fitting. However, since this region lies right next to the Ta 4f peak region, a dummy peak (gray) was fitted into the first peak of the Ta 5p region to account for the residual signal. (a5, b5) Weighted Ta^5+^:Ta^0^ peak ratios for all angles and peak regions, fitted by eq 1. Since the EALs for Ta and Ta_2_O_5_ are interdependent and defined by the kinetic energy of the photoelectrons, only the EAL of Ta_2_O_5_ is representatively plotted on the axis. (a6, b6) Ta^5+^ depth profiles, corresponding to *d* and σ obtained from the fit in (a5, b5), respectively. The Ta^5+^ number densities were normalized to their respective nominal maximum values.

Figure 2c shows the change of thickness of the upper Ta_2_O_5_ layer, the metallic Ta, as well as the total thickness of the Ta layers, including the metallic Ta and the Ta_2_O_5_ layers above and below, as a function of the applied gate voltage. A threshold voltage of approximately −1.0 V is required to oxidize Ta. Above this voltage, the average Ta_2_O_5_ thickness increases linearly with the applied gate voltage, as expected,^25^ reaching a thickness of (3.9 ± 0.3) nm after −3.0 V gating. In parallel, the Ta thickness reduces from (14.5 ± 0.1) to (13.1 ± 0.1) nm. This is notably less compared to the increase in the Ta_2_O_5_ thickness. The reason is the significantly lower mass density of Ta_2_O_5_ compared to metallic Ta. The total thickness therefore increases.

Figure 2d presents the evolution of the interface width at the upper Ta_2_O_5_/Ta interface with increasing gate voltage. In XRR simulations, the interface width corresponds to the fitted roughness value. However, such broadening can originate either from topographical roughness or from a gradual concentration gradient across the interface, which are virtually indistinguishable in specular XRR. Therefore, we refer to this parameter more generally as the interface width. Overall, the extracted interface width remains very small, especially when compared to the layer thickness. At low gate voltages, the XRR-derived oxide thickness and interface width are effective parameters describing a gradual oxidation profile rather than layer-by-layer growth of a fully developed Ta_2_O_5_. During the initial gating, the data suggests a slight increase in interface width. At higher gate voltages, it appears to level off or even decrease slowly, which, however, remains within the experimental uncertainty and requires further confirmation. The initial increase likely originates from the structural reorganization of the topmost Ta layer and the density change accompanying the formation of the first Ta_2_O_5_ layer. The subsequent leveling, or possibly slight decrease, indicates smooth and uniform growth of the additional oxide layers. Overall, the interface width changes by only about (0.1 ± 0.05) nm, which is less than the thickness of a single Ta monolayer (the metal atomic radius of Ta is 0.14 nm^24^).

To assess the influence of gate voltage, gating time, and the stability and reversibility of the induced oxidation, Figure 2e shows the evolution of the longitudinal resistance during consecutive gating steps and over time. The longitudinal resistance reflects the amount of conductive Ta and thus serves as an estimate of the degree of oxidation. This method enables immediate measurements on the same holder as used for IL gating—starting the measurement typically within 3 min after switching off the gate voltage—without removing the IL or top electrode.

With increasing gate voltage (red region in Figure 2e), the resistance follows the same trend as the Ta_2_O_5_ thickness obtained from XRR (blue open circles), confirming the correspondence between the two methods. Changes induced by longer gating times (repeated −2.0 V steps) are significantly smaller than those from increasing the gate voltage. This is why we focused on voltage variation in this study. Although the additional oxidation decreases with repeated gating, full saturation is not reached within a total of 50 min of gating.

The oxidation state remains highly stable after gating. Both short-term monitoring over 15 h (blue region in Figure 2e) and a long-term control measurement after 2 months (Figure 2f) show negligible changes. The XRR measurements shown in Figure 2f also confirm that neither several hours of X-ray exposure during XPS nor the exposure to atmosphere alter the thickness and roughness of the layers. Finally, reversibility tests (yellow region in Figure 2e) reveal that the oxidation cannot be recovered by reversing the gate voltage from −2.0 to +2.0 V. Only at the maximum accessible voltage of +3.0 V, a marginal decrease in resistance is observed, which can be reversed again by applying a negative voltage of −2.0 V.

To obtain more information about the chemical processes during IL gating and to validate the results from XRR, we performed XPS measurements. The long-term stability of the oxidation was confirmed by repeating the XPS measurement of the sample with 2 nm HfO_2_ gated at −2.0 V after 5 months. By analyzing the spectra obtained at different emission angles (angle-resolved XPS) as well as peak regions (multiple-energies approach), information on the depth distribution of the elements can be obtained (see Figure 1c and reference 26). Therefore, we fitted all four available Ta peak regions, Ta 4f, Ta 5s, Ta 4d and Ta 4p at six different emission angles, 0, 15, 25, 35, 45, and 55° relative to the sample normal. For larger angles, the error on the EAL, which determines the probability that a photoelectron reaches the sample surface without scattering, becomes significantly larger.^27^ Therefore, no angles larger than 55° were considered. The results from XRR were used as starting parameters for the fitting of all XPS peaks. More details on the fit parameters and procedure can be found in SI 3–5.

Figure 3 shows a subset of the fitted data for the sample with 2 nm HfO_2_ capping. To investigate the effect of gating, the spectrum of the as-deposited state (Figure 3a) is compared to the state after applying the maximum gate voltage of −3.0 V for 10 min (Figure 3b). The survey spectra in Figure 3a1,b1 display the full energy range containing Ta and Hf peaks (survey spectra showing the complete measured energy range can be found in SI 6). The three different energy regions with the clearest chemical shift between the metallic Ta (Ta^0^, green) and oxidized Ta_2_O_5_ (Ta^5+^, blue) component peaks are shown in the close-ups below the surveys (Figure 3a2–4 and b2–4). In the as-deposited state, only the metallic Ta^0^ and Hf^4+^ (HfO_2_, purple) show pronounced peaks. Almost no oxidized Ta^5+^ is present. In contrast, after gating at −3.0 V for 10 min, the Ta^0^ peaks get significantly smaller while Ta^5+^ now shows large, distinct peaks, confirming a significant oxidation of the Ta layer. Notably, no additional peaks from intermediate Ta oxidation states are visible. Such peaks would be expected between the Ta^0^ and Ta^5+^ peaks, as was, for example, observed at the interface between Si and SiO_2_.^28^ As none of the gating steps show such peaks (see SI 7), we conclude that an atomically sharp interface from Ta to Ta_2_O_5_ forms and propagates upon gating. This also matches the XRR results, which showed a sharp interface at all gate voltages.

When examining the HfO_2_ spectra, it is noteworthy that no metallic Hf^0^ peaks appear after gating. This indicates that the Hf–O bonds remain intact, consistent with the high energetic cost of bond dissociation. Possible oxygen sources therefore include interstitial or defect-bound oxygen within the HfO_2_ layer, oxygen dissolved in the IL, or atmospheric oxygen entering from ungated edge regions of the HfO_2_. Prior work by An et al. demonstrated the supply of oxygen ions from a comparable IL into adjacent metallic layers by doping the IL with H_2_^18^O and tracking the migration of the ^18^O under gating.^29^ Based on this evidence, oxygen supplied by the IL is likely the dominant source in the present system.

The applied electric field transports these O^2–^ anions inward through the dielectric stack. The following Ta oxidation process is likely comparable to atmospheric passivation, for which a self-generated electric field—the so-called Mott potential—drives ion motion. The Mott potential is of comparable magnitude to the electric field required for IL gating, on the order of 10^6^–10^7^ V/cm.^30,31^ As the oxide thickens, this electric field progressively weakens until it can no longer sustain either the required electron tunneling or ion transport, resulting in an equilibrium oxide thickness.^30^ Depending on the defect chemistry, many passivation models assume that the metal cation migration can play a dominant role in oxide growth.^25,30^ However, since no intermixing of Hf^4+^ and Ta^5+^ is detected at the HfO_2_/Ta_2_O_5_ interface (Figure S7), Ta^5+^ migration through the HfO_2_ layer can be excluded. Consequently, oxygen can be identified as the primary mobile species, migrating inward through the HfO_2_ and Ta_2_O_5_ as anions or outward as oxygen vacancies.

Another noteworthy change after gating is the appearance of sharp indium peaks (In^3+^, red) in the spectrum of Ta 4p and Hf 4p (Figure 3b2–4). Since this In is not present in the as-deposited state, it has to originate from the ITO coating of the glass plate, which is used as the top gate contact. Apparently, some of this In is released from the ITO during gating. The standard electrode potential for the In^3+^/In^0^ redox couple is −0.34 V versus the standard hydrogen electrode, which is lower than the applied voltage. Therefore, likely In^3+^ is reduced to In^0^, and deposits on the sample surface. After gating, it oxidizes back to In^3+^. The intensity of the In peaks does not significantly change for different emission angles, in contrast to the Ta peaks, which become significantly smaller for larger angles. Even the adjacent Hf peak decreases slightly in size for increasing angles in contrast to the In peaks. Hence, the In atoms were deposited only at the surface of the sample and did not penetrate the HfO_2_.

Based on the finding that there are no spectroscopically resolvable intermediate oxidation states at the interface between Ta and Ta_2_O_5_, we can model the interface assuming a symmetric transition between Ta and Ta_2_O_5_ described by an error function. This is the common assumption for metallic interfaces, as also applied in XRR. The thickness *d* of the Ta_2_O_5_ layer and width σ of the interface between Ta and Ta_2_O_5_ can then be calculated using eq 1.

$$\begin{array}{l} \frac{I_{{\mathrm{Ta}}^{5+}}N_{{\mathrm{Ta}}^{0}}}{I_{{\mathrm{Ta}}^{0}}N_{{\mathrm{Ta}}^{5+}}}\left(L_{{\mathrm{Ta}}_{2}O_{5}},L_{\mathrm{Ta}},\varphi \right) \end{array}=\frac{L_{{\mathrm{Ta}}_{2}O_{5}}}{L_{\mathrm{Ta}}}\exp\left(\frac{d}{L_{{\mathrm{Ta}}_{2}O_{5}}\cos\varphi }-\frac{d}{L_{\mathrm{Ta}}\cos\varphi }\right)\cdot \frac{\mathrm{erfc}\left(-\frac{d}{\sqrt{2}\sigma }\right)-\mathrm{erfc}\left(\frac{\sigma }{\sqrt{2}L_{{\mathrm{Ta}}_{2}O_{5}}\cos\varphi }-\frac{d}{\sqrt{2}\sigma }\right)\exp\left(\frac{\sigma^{2}}{{2\left(L_{{\mathrm{Ta}}_{2}O_{5}}\cos\varphi \right)}^{2}}-\frac{d}{L_{{\mathrm{Ta}}_{2}O_{5}}\cos\varphi }\right)}{\mathrm{erfc}\left(\frac{d}{\sqrt{2}\sigma }\right)+\mathrm{erfc}\left(\frac{\sigma }{\sqrt{2}L_{\mathrm{Ta}}\cos\varphi }-\frac{d}{\sqrt{2}\sigma }\right)\exp\left(\frac{\sigma^{2}}{{2\left(L_{\mathrm{Ta}}\cos\varphi \right)}^{2}}-\frac{d}{L_{\mathrm{Ta}}\cos\varphi }\right)}$$ 1

Thereby, $\frac{I_{{\mathrm{Ta}}^{5+}}N_{{\mathrm{Ta}}^{0}}}{I_{{\mathrm{Ta}}^{0}}N_{{\mathrm{Ta}}^{5+}}}$ denotes the normalized intensity ratio of the metallic and corresponding oxidized Ta peak within the same peak region. The peak intensities *I*_Ta^5+^_ and *I*_Ta^0^_ of the oxidized and metallic Ta peaks are normalized to the atomic number densities *N*_Ta^5+^_ and *N*_Ta^0^_ of Ta^5+^ and Ta^0^ atoms per unit volume of the pure compounds, respectively. The intensity ratio depends on the emission angle φ, as well as on the EALs *L*_Ta_2_O_5__ and *L*_Ta_ in Ta_2_O_5_ and Ta, respectively, which are functions of the kinetic energy *E*_kin_ of the photoelectrons of the respective peak region. More details on the equation and the required parameters can be found in SI 3 as well as in references 26 and 32.

For all peak regions, the normalized intensity ratios have to correspond to the same values of *d* and σ. Therefore, all fits were optimized accordingly. Figure 3a5 and b5 show the results of the weighted intensity ratios and the fit of eq 1 as a function of φ and *L*_Ta_2_O_5__ (*E*_kin_). From the corresponding results of the fit for *d* and σ, we obtained the Ta^0^ and Ta^5+^ depth profiles (Figure 3a6 and b6). Both *d* and σ were thereby consistent with the XRR results, such that the values obtained from both methods are identical.

Figure 4a shows the comparison between the SLD profile obtained from XRR (SLD-XRR) and the depth profiles obtained from XPS (SLD-XPS), given by the depth-*z* dependent number densities $N\left(Ta^{0}\right)=\frac{N_{Ta^{0}}}{2}\mathrm{erfc}\left(\frac{d-z}{\sqrt{2}\sigma }\right)$ and $N\left(Ta^{5+}\right)=\frac{N_{Ta^{5+}}}{2}\mathrm{erfc}\left(\frac{z-d}{\sqrt{2}\sigma }\right)$ , after gating at −3.0 V. When normalized and expressed in percent, *N*(Ta^5+^) is identical to the Ta^5+^ depth profile, as plotted in Figure 4b,c. For a more direct comparison between the results from XRR and XPS, the SLD-XPS profile was calculated from *N*(Ta^0^) and *N*(Ta^5+^) at the energy of Cu Kα radiation. Details on the calculation can be found in SI 8. In XPS, we only investigated the interface between Ta and Ta_2_O_5_, so the SLD-XPS was only calculated for that interface. The SLD profiles obtained from XPS and XRR are almost perfectly congruent at that interface. This reflects that both methods model the interface as an error function, and yield the same average interface position and width. To focus on the Ta_2_O_5_/Ta interface, we will use the Ta^5+^ depth profiles from XPS in the following to discuss the evolution of interface position and width under gating, as well as the effect of HfO_2_ thickness.

**Figure 4:**
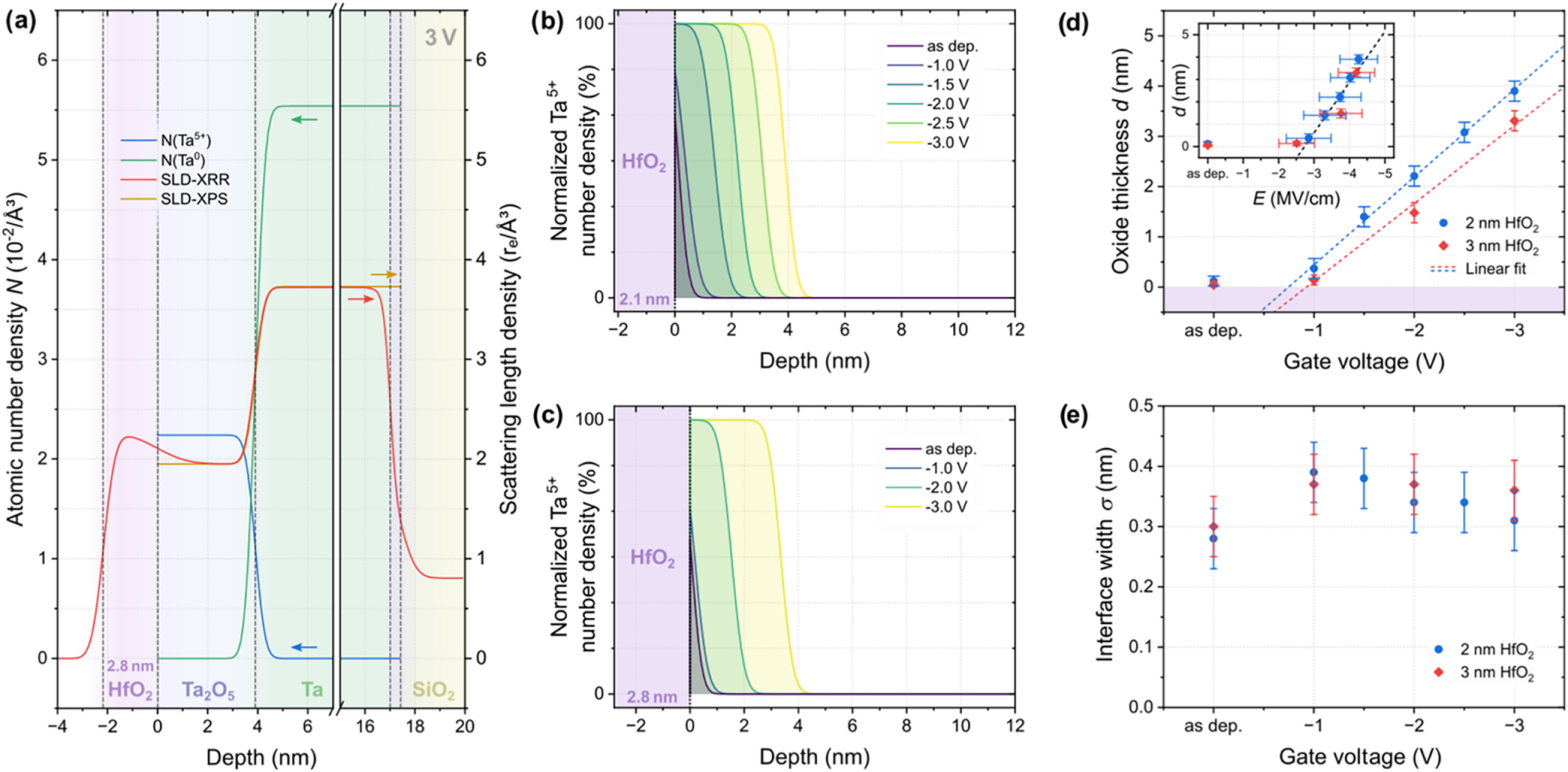
(a) Comparison of the scattering length density (SLD) as obtained from XRR and the depth profiles as well as corresponding SLD (calculated for the energy of Cu Kα radiation to compare to XRR, details can be found in SI 8) as obtained from XPS for the sample with 2 nm HfO_2_ capping and gated at −3.0 V. (b, c) Ta^5+^ depth profiles for the samples with nominal (b) 2 nm and (c) 3 nm HfO_2_ capping as a function of the gate voltage. (d) Average Ta_2_O_5_ layer thicknesses obtained from the depth profiles in (b) and (c) as a function of the gate voltage. The inset shows the same thicknesses as a function of the applied electric field (assuming an electric double layer of the IL of 1 nm, added up with the HfO_2_ and Ta_2_O_5_ thicknesses). The error bar reflects the difference resulting from assuming an electric double-layer thickness of 2 nm. (e) Interface width at the Ta_2_O_5_/Ta interface as a function of the gate voltage as obtained from the depth profiles in (b, c).

The Ta^5+^ depth profiles are depicted in Figure 4b,c as a function of the gate voltage, for two different HfO_2_ thicknesses. The results are qualitatively the same for both HfO_2_ thicknesses, showing a sharp interface between Ta and Ta_2_O_5_, which is pushed deeper into the Ta layer with increasing gate voltage. Figure 4d shows the comparison of the corresponding average oxide layer thicknesses *d* for all samples. For both HfO_2_ thicknesses, the threshold voltage required to significantly oxidize the Ta is around −1.0 V. Above this voltage, the average oxide layer thickness increases linearly with the applied gate voltage in the investigated range. Overall, the oxide layer thickness is larger for all gate voltages when a thinner HfO_2_ capping of only 2 nm is used. This is most likely due to the larger electric field created by applying an identical gate voltage over a thinner dielectric material.

In the inset (Figure 4d), the average oxide layer thickness is therefore plotted as a function of the electric field, calculated by assuming an IL electric double layer thickness of 1 nm. This value corresponds approximately to the combined thickness of one negatively and one positively charged ion layer, across which the voltage drop in the IL occurs. The relation between *d* and *E* is very similar for both HfO_2_ thicknesses, with the same initial threshold electric field of ≈−2.8 MV/cm to start oxidizing the Ta. Remarkably, the required electric field increases by ≈−0.45 MV/cm for every additional nm of Ta_2_O_5_. This shows that the threshold electric field needed to drive oxygen ions from HfO_2_ to the Ta_2_O_5_/Ta interface is not constant—as might be expected—but grows with oxide thickness. Even when considering the saturated film thickness, which can be estimated from Figure 2e to be approximately 2.9 nm for the −2.0 V gating step, the required electric field still increases with oxide thickness. One possible explanation is the self-passivating nature of Ta, for which the energetic cost of further oxidation increases with depth.^32^ This is also consistent with temperature-dependent oxide growth reported in the literature.^33^ A voltage-dependent increase in the IL’s electric double-layer thickness might also contribute, but would likely be too small to account for the observed slope.

The Ta_2_O_5_/Ta interface width shows very similar behavior regardless of the HfO_2_ thickness (Figure 4e). For both samples, an initial slight increase is observed, followed by leveling or a possible minor decrease at higher gate voltages. Importantly, the interface remains extremely sharp, with a width of only a few Å.

## Conclusion

Our results demonstrate that oxygen ions can be driven several nm into metallic films. In Ta, penetration depths up to 4 nm were achieved by applying −3 V for 10 min. This depth exceeds the typical thickness of magnetic thin films, which are usually 0.8–0.9 nm for out-of-plane magnetized layers and 1.0–2.5 nm for in-plane layers. Spacer layers, such as in synthetic antiferromagnets or magnetic tunnel junctions, can be even thinner. Thus, the observed oxygen penetration depth is sufficient to alter the properties of buried layers or interfaces. Depending on the application, this effect can be exploited deliberately or must be carefully managed when preservation of lower interfaces is required.

Furthermore, we find that the Ta_2_O_5_/Ta interface propagates linearly with applied voltage into the Ta film. The required electric field between IL and Ta electrode is of comparable strength as the Mott potential in passivation processes. Our results demonstrate that oxygen anions, rather than metallic cations, are the primary mobile species underlying the oxide growth. Notably, the interface remains atomically sharp (few Å) at all voltages, indicating sequential formation of complete Ta_2_O_5_ layers. This behavior is consistent with electric-field screening. The sharpness of the interface further shows that oxidation occurs uniformly across the analyzed sample region, which reflects the high uniformity of the sputtered Ta film. In other materials, additional effects such as grain boundary diffusion may play a role. For example, Gilbert et al. reported that solid-state gating in Pd/Co/AlO_*x*_/GdO_*x*_ stacks leads to two distinct magnetic phases.^34^ They attributed this to preferential oxygen transport along grain boundaries combined with slower diffusion within grains. In such cases, the process inside each grain or homogeneous region will likely resemble the behavior identified in this study. Thus, our findings provide a framework to interpret oxide depth profiles and disentangle competing physical effects in a broad range of systems.

When choosing the thickness of the dielectric capping layer, it is important to note that thinner HfO_2_ enhances oxidation by increasing the electric field. Still, for reliable IL gating, a continuous capping layer is essential. The minimum thickness is therefore limited by the width of the underlying interface and growth characteristics of the capping layer.

Finally, we observe the migration of indium atoms from the ITO electrode onto the sample surface during gating. This is important to consider for all applications requiring a well-defined surface, e.g., when postprocessing the sample after removal of the IL and gate.

The results from XRR and XPS showed very good agreement, thereby validating the derived depth profiles. In the present case, XRR provided depth profiles with lower uncertainty while requiring less experimental effort. However, the uncertainty of the extracted depth profiles is material-dependent, and the relative advantages of the two techniques may shift for different systems. Specifically, XRR yields more reliable results in cases of pronounced electron density contrasts, while XPS achieves higher sensitivity when several well-separated emission peaks with large chemical shifts are present. Beyond depth profiling, the evaluation of the full XPS spectrum using the multiple-energies approach provided valuable insights into oxidation states and enabled the detection of indium on the sample surface. Hence, the two techniques offer complementary strengths for a more robust assessment of interfacial structure and composition.

The presented results elucidate the mechanism of oxygen migration and Ta_2_O_5_ formation during IL gating, providing quantitative design rules for magneto-ionic stacks. This understanding is key for advancing low-power nanoelectronic, spintronic and neuromorphic devices.

## Experimental Section

### Sample Growth

The samples were grown on p-doped thermally oxidized Si/SiO_2_ (100 nm SiO_2_) at room temperature in an industrial Singulus Rotaris magnetron sputtering tool. The base pressure was 5 × 10^–8^ mbar. As a metallic film, Ta was grown using DC magnetron sputtering. HfO_2_ was grown using RF magnetron sputtering, without introducing additional oxygen, in pure Ar atmosphere at a pressure of 6 × 10^–3^ mbar. In this way, a very high sample quality and homogeneity was achieved for both layers, as evidenced by the smooth growth observed by XRR. All samples with the same nominal HfO_2_ thickness were grown together on the same wafer and afterward cut into 5 × 8 mm^2^ large pieces.

### IL Gating

The IL 1-ethyl-3-methylimidazolium-bis­(trifluoromethylsulfonyl)-imide ([EMIM]^+^ [TFSI]^−^) (CAS: 174899–82–2, SKU: 711691–100G, >98% (HPLC), from Sigma-Aldrich) was used to apply the gate voltages. A drop of the IL was placed in each corner of the HfO_2_ surface. A glass slide (area 5 × 7 mm^2^) coated with ITO, floating on top of the IL, was used as the top contact for the gate voltage. To ground the Ta, serving as the bottom contact, the Ta was wirebonded through the insulating layers on top. By applying negative gate voltages to this setup, oxygen ion species can be moved from HfO_2_ into the Ta. Gate voltages between −1.0 and −3.0 V were applied at room temperature using a Keithley 2400. Thereby, the voltage was slowly increased, to minimize instabilities, up to the target voltage which was applied for 10 min. Afterward, the IL was washed off in acetone.

### XRR Measurements

XRR measurements were performed using a Bruker D8 X-ray diffractometer using Cu Kα radiation. The primary beam was focused using a line focus of 12 mm length, followed by a Göbel mirror and a slit with a nominal width of 0.6 mm. The resulting X-ray beam incident on the sample is approximately rectangular with dimensions 12 mm × 0.6 mm. The secondary beam path had two parallel slits with a width of 0.6 mm each, to filter out off-specular X-rays. The geometric acceptance angle at the detector is approximately ± 0.05°. The XRR data was measured in the 2θ-range from 0.3 to 10° with a step size of 0.01° and a collection time of 1.0 s per step. The resulting instrument resolution is of the order of the step size. All densities, the HfO_2_ thickness and the thickness and interface width of the bottom Ta_2_O_5_ layer were fixed in the XRR analysis. Detailed information on the reasons and implications can be found in SI 1. The analysis was done using the software GenX version 3.7.4. All data was measured in a specular geometry. Therefore, the spec_nx mode of the advanced reflectivity plugin was used, which fits the data using the Parratt algorithm.^35,36^ The interface width is defined as the root-mean-square roughness at the top interface of the layer, assuming a Gaussian deviation from the ideal interface.

### XPS Measurements

XPS measurements were performed using an ESCA-unit Phi 5000 VersaProbe III photoelectron spectrometer. A monochromated raster scanned micro focused X-ray beam with Al Kα radiation (1486.6 eV) and 25 W at 15 kV was used. The resulting analyzed area has a diameter of 100 μm. A 180° hemispherical electron energy analyzer with a scanning input lens was used, which is synchronized to the scanning X-ray beam. It has a work function of 4.39 eV and was positioned at a fixed angle of 45° toward the X-ray source. The energy resolution of the instrument, defined as the full width at half-maximum (fwhm) of the Ag 3d_5/2_ peak, is 0.7 at 55 eV pass energy. For each sample, a survey (pass energy 224 eV) as well as the O 1s and Ta 4p, 4d, 5s and 4f energy ranges (pass energy 55 eV) were recorded for electron emission angles φ between 0 and 55° relative to the surface normal by tilting the sample. The surveys were measured from the Fermi edge up to 1300 eV in steps of 0.4 eV and the individual peak regions were scanned in steps of 0.1 eV. The binding-energy scale was referenced to the Fermi-edge cutoff of the valence band.^37^ The analysis was done using the software CasaXPS version 2.3.26PR1.0.

## Supplementary Material



## Data Availability

Data that support the findings of this study are openly available in Zenodo at https://doi.org/10.5281/zenodo.18008337.^38^

## Notes

XRR and XPS measurements, tables with the used material constants, list of peak positions and parameters for XPS peak fits and further information on the equations used for the analysis (PDF)
