# Supplementary material for: Depth Profiling of Oxygen Migration in Ta/HfO2 Stacks during Ionic Liquid Gating
Source: ACS Appl Mater Interfaces. 2026 Jan 13;18(3):6200–9. doi: 10.1021/acsami.5c22179 (PMC12862756; doi:10.1021/acsami.5c22179)
Supplement: Supplementary file 1 [file am5c22179_si_001.pdf]

Supporting Information (SI)

Depth Profiling of Oxygen Migration in Ta/HfO_2 Stacks During Ionic Liquid Gating

Beatrice Bednarz,[1,‡,*] Martin Wortmann,[2,‡] Olga Kuschel,[1,3] Fabian Kammerbauer,[1] Mathias Kläui,[1] Andreas Hütten,[2] Joachim Wollschläger,[3] Gerhard Jakob,[1] and Timo Kuschel[1,2,*]

[1)] *Institute of Physics, Johannes Gutenberg University Mainz, Staudingerweg 7, 55128 Mainz, Germany*
[2)] *Faculty of Physics, Bielefeld University, Universitätsstraße 25, 33615 Bielefeld, Germany*
[3)] *Faculty of Physics, Osnabrück University, Barbarastraße 7, 49076 Osnabrück, Germany*

‡ Equal author contribution
* Correspondence: bbednarz@uni-mainz.de, tkuschel@uni-mainz.de

SI 1. X-ray reflectivity (XRR) fit parameters

The XRR analysis was performed in GenX[1] (homepage: https://aglavic.github.io/genx/) version 3.7.4, using the following slab model: SiO_2 / Ta_2O_5 bottom / Ta / Ta_2O_5 top / HfO_2.

For all layers, literature densities were used. For HfO_2 and the top Ta_2O_5, the reason is their very similar mass density and correspondingly similar scattering length density (SLD). Therefore, they are nearly indistinguishable in XRR, which leads to unreasonable density values when fitted freely. For Ta, a free fit gave densities very close to the literature density (within 0.3 g/cm^3), which, however, differed between the samples after the different gating steps. Since all samples with the same HfO_2 thickness were sputter-deposited together on the same wafer, their Ta densities cannot be different. Therefore, the literature density was set for all samples. For the bottom Ta_2O_5 layer, the thickness is too thin to fit the density freely.

Additionally, for the HfO_2 layer and the bottom Ta_2O_5 layer, the thicknesses were fixed according to their thickness in the corresponding as-deposited sample. For the bottom Ta_2O_5, the underlying assumption is that the Ta layer above screens the electric field and therefore the bottom Ta_2O_5 layer is not significantly affected by the gating. Also, the interface width of this layer was fixed according to the as-deposited state, using the knowledge that the roughness of the SiO_2 wafer is in the range of 1-3 Å. For HfO_2, the reason to fix the thickness is the indistinguishability between the HfO_2 and the top Ta_2O_5 layer because of their similar densities. Using the knowledge from XPS, that the HfO_2 does not get reduced to metallic Hf (no Hf^0 peak and also no shoulder or other sign of a change due to gating appears in XPS), the assumption that the HfO_2 layer remains unaffected by gating seems justified. Still, this uncertainty was included in the error on the Ta_2O_5 thickness (see error discussion below).

Table S1 shows the parameters fixed in the XRR fit. Tables S2 and S3 provide the corresponding results for the layer thickness d and interface width σ for the samples with 2 and 3 nm HfO_2, respectively. Figure S1 shows the corresponding fits and SLD profiles for all samples.

Tables S2 and S3 also give the estimated uncertainties for all values. For the layer thicknesses, the uncertainty of the fits is estimated to be 0.1 nm. Additionally, the HfO_2 layer has an uncertainty due to the assumption that it is unaffected by gating. It was estimated to be 0.1 nm for all gating steps, leading to a total uncertainty on the HfO_2 thickness of 0.14 nm. This error gets propagated to the thickness of the upper Ta_2O_5 layer because of their similar densities, additionally to the fitting uncertainty. For the interface width, the basic fitting uncertainty is estimated to be 0.05 nm. However, toward the sample surface (for HfO_2) and the substrate (Ta_2O_5 bottom) the uncertainty is larger and estimated to be 0.1 nm. At the HfO_2/Ta_2O_5 interface, the error is significantly larger because of the similar densities.

Table S1: Fixed parameters of XRR fits. Densities taken from literature values given by Merck KGaA and Kurt J. Lesker Company are used.

		HfO_2	Ta_2O_5 top	Ta	Ta_2O_5 bottom	SiO_2
	ρ (g/cm^3)	9.7	8.2	16.7	8.2	2.7
Ta (15)/ HfO_2 (2)	d (nm)	2.14			0.4	
	σ (nm)				0.2	
	ρ (g/cm^3)	9.7	8.2	16.7	8.2	2.7
Ta (15)/ HfO_2 (3)	d (nm)	2.84			0.4	
	σ (nm)				0.3	

Table S2: Fitted parameters from XRR for the samples Si/ SiO_2/ Ta (15)/ HfO_2 (2) for all applied gate voltages. The values of the interface width σ, which can be caused either by topographical roughness or a gradual concentration gradient, are defined at the upper interface of each layer. The thickness d of the Ta_2O_5 layer and interface width σ at the Ta_2O_5/Ta interface, which are particularly discussed in the manuscript, are highlighted with a green background. The gray values were fixed (see table S1) and are given here together with their estimated uncertainties. SiO_2 is not included because the substrate is assumed to have infinite thickness in XRR. The substrate interface width was fixed to the range 1–3 Å, which is the topographical roughness of the substrates.

		HfO_2	Ta_2O_5 top	Ta	Ta_2O_5 bottom
As deposited	d (nm)	2.14 ± 0.10	0.12 ± 0.10	14.5 ± 0.1	0.4 ± 0.1
	σ (nm)	0.4 ± 0.1	0.12 ± 0.05	0.28 ± 0.05	0.2 ± 0.1
-1.0 V	d (nm)	2.14 ± 0.14	0.4 ± 0.2	14.4 ± 0.1	0.4 ± 0.1
	σ (nm)	0.4 ± 0.1	0.4 ± 0.2	0.39 ± 0.05	0.2 ± 0.1
-1.5 V	d (nm)	2.14 ± 0.14	1.4 ± 0.2	14.0 ± 0.1	0.4 ± 0.1
	σ (nm)	0.5 ± 0.1	0.5 ± 0.3	0.38 ± 0.05	0.2 ± 0.1
-2.0 V	d (nm)	2.14 ± 0.14	2.2 ± 0.2	13.7 ± 0.1	0.4 ± 0.1
	σ (nm)	0.5 ± 0.1	0.4 ± 0.3	0.34 ± 0.05	0.2 ± 0.1
-2.5 V	d (nm)	2.14 ± 0.14	3.1 ± 0.2	13.4 ± 0.1	0.4 ± 0.1
	σ (nm)	0.4 ± 0.1	0.6 ± 0.5	0.34 ± 0.05	0.2 ± 0.1
-3.0 V	d (nm)	2.14 ± 0.14	3.9 ± 0.2	13.1 ± 0.1	0.4 ± 0.1
	σ (nm)	0.4 ± 0.1	1.0 ± 0.7	0.31 ± 0.05	0.2 ± 0.1

Table S3: Fitted parameters from XRR for the samples Si/ SiO_2/ Ta (15)/ HfO_2 (3) at all applied gate voltages, presented equivalently to table S2 for the samples with 2 nm HfO_2.

		HfO_2	Ta_2O_5 top	Ta	Ta_2O_5 bottom
As deposited	d (nm)	2.84 ± 0.10	0.05 ± 0.05	14.5 ± 0.1	0.4 ± 0.1
	σ (nm)	0.4 ± 0.1	0.07 ± 0.05	0.30 ± 0.05	0.3 ± 0.1
-1.0 V	d (nm)	2.84 ± 0.14	0.14 ± 0.10	14.5 ± 0.1	0.4 ± 0.1
	σ (nm)	0.4 ± 0.1	0.2 ± 0.1	0.37 ± 0.05	0.3 ± 0.1
-2.0 V	d (nm)	2.84 ± 0.14	1.5 ± 0.2	14.0 ± 0.1	0.4 ± 0.1
	σ (nm)	0.3 ± 0.1	0.4 ± 0.2	0.37 ± 0.05	0.3 ± 0.1
-3.0 V	d (nm)	2.84 ± 0.14	3.3 ± 0.2	13.2 ± 0.1	0.4 ± 0.1
	σ (nm)	0.4 ± 0.1	0.6 ± 0.4	0.36 ± 0.05	0.3 ± 0.1

SI 2. Overview over all XRR fits

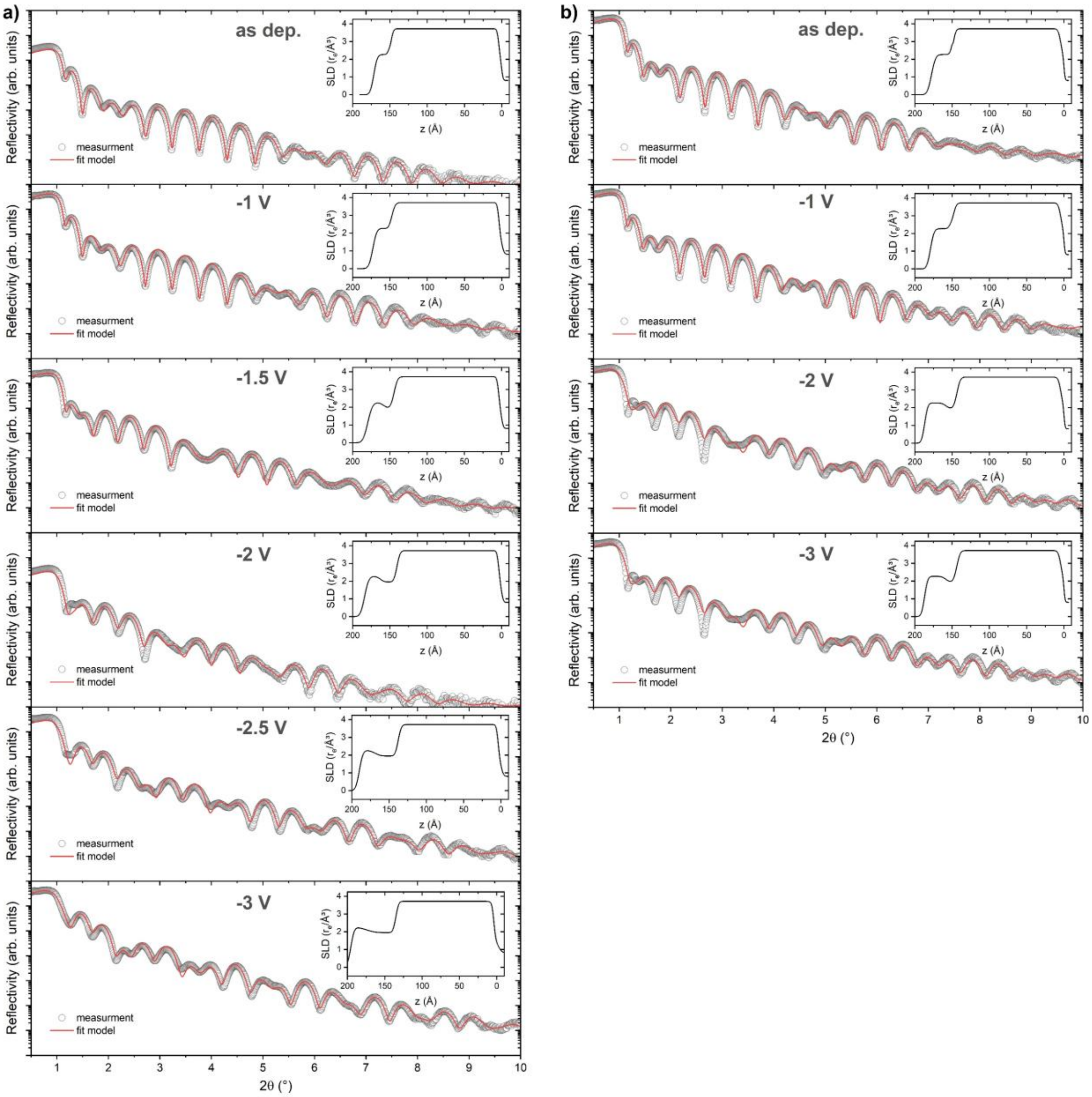

Figure S1: XRR fits and corresponding SLDs for the samples with (a) 2 and (b) 3 nm HfO_2, respectively, at all gate voltages. The narrow oscillation corresponds to the Ta thickness, while the wider oscillation with the visibly decreasing period corresponds to the combined thickness of HfO_2 and the top Ta_2O_5.

SI 3. Origin of the equation used to obtain d and σ from the XPS data

In XPS, the binding energy of the emitted photoelectrons provides information on the corresponding element and its oxidation state. The number of photoelectrons ΔI, which reach the surface without inelastic scattering, can be expressed according to the Beer-Lambert law, as

$$\Delta I = \Delta I_0 \exp\left(\frac{-z}{L\cos(\varphi)}\right) \quad \text{with} \quad \Delta I_0 \propto \Delta z \tag{S1}$$

with the excited number of photoelectrons ΔI_0 created in a thin layer with thickness Δz at a depth z below the surface, the distance $z/\cos(\varphi)$ traveled by the photoelectrons emitted at an angle φ toward the sample normal and the inelastic mean free path (IMFP) or effective attenuation length (EAL) L of the material (an explanation of the difference between the IMFP and EAL can be found in SI 4). In this study, we consider a two-layer system with a Ta_2O_5 layer with an average thickness d on top of a Ta film. We assume a gradual interface with a width σ between the two layers, which can be approximated by a complementary error function $\mathrm{erfc}\left(\frac{z-d}{\sqrt{2}\,\sigma}\right)$. In this case, the number of photoelectrons from the Ta_2O_5 layer $I_{\mathrm{Ta}^{5+}}$ and from the Ta layer $I_{\mathrm{Ta}^{0}}$ can be expressed as

$$\Delta I_{\mathrm{Ta}^{5+}} = \Delta I_{0,\mathrm{Ta}^{5+}} \exp\left(\frac{-z}{L_{\mathrm{Ta_2O_5}}\cos(\varphi)}\right) \tag{S2}$$

with $\Delta I_{0,\mathrm{Ta}^{5+}} \propto N_{\mathrm{Ta}^{5+}} \Delta z \,\mathrm{erfc}\left(\frac{z-d}{\sqrt{2}\,\sigma}\right)$

$$\Delta I_{\mathrm{Ta}^{0}} = \Delta I_{0,\mathrm{Ta}^{0}} \exp\left(\frac{-(z-d)}{L_{\mathrm{Ta}}\cos(\varphi)}\right) \exp\left(\frac{-d}{L_{\mathrm{Ta_2O_5}}\cos(\varphi)}\right) \tag{S3}$$

with $\Delta I_{0,\mathrm{Ta}^{0}} \propto N_{\mathrm{Ta}^{0}} \Delta z \,\mathrm{erfc}\left(\frac{d-z}{\sqrt{2}\,\sigma}\right)$

Thereby, $\Delta I_{0,\mathrm{Ta}^{5+}}$ and $\Delta I_{0,\mathrm{Ta}^{0}}$ are the excited number of photoelectrons reaching the detector from a thin layer with thickness Δz in the oxide and metal, respectively, $L_{\mathrm{Ta_2O_5}}$ and L_{Ta} are the EALs of the oxide and the metal and $N_{\mathrm{Ta}^{5+}}$ and $N_{\mathrm{Ta}^{0}}$ denote the number densities of oxidized or metallic Ta atoms, respectively. Integrating these two equations from $0 \le z \le \infty$ (assuming that the thickness of the Ta layer is much thicker than L_{Ta}) yields the respective XPS peak intensities $I_{\mathrm{Ta}^{5+}}$ and $I_{\mathrm{Ta}^{0}}$. By calculating the quotient of the two and rearranging, we obtain equation (1) from the main text

$$\frac{I_{\mathrm{Ta}^{5+}} \cdot N_{\mathrm{Ta}^{0}}}{I_{\mathrm{Ta}^{0}} \cdot N_{\mathrm{Ta}^{5+}}}\left(L_{\mathrm{Ta_2O_5}}, L_{\mathrm{Ta}}, \varphi\right)$$

$$= \frac{L_{\mathrm{Ta_2O_5}}}{L_{\mathrm{Ta}}} \cdot \exp\left(\frac{d}{L_{\mathrm{Ta_2O_5}}\cos\varphi} - \frac{d}{L_{\mathrm{Ta}}\cos\varphi}\right) \cdot \frac{\mathrm{erfc}\left(-\frac{d}{\sqrt{2}\,\sigma}\right) - \mathrm{erfc}\left(\frac{\sigma}{\sqrt{2}\,L_{\mathrm{Ta_2O_5}}\cos\varphi} - \frac{d}{\sqrt{2}\,\sigma}\right) \cdot \exp\left(\frac{\sigma^2}{2\left(L_{\mathrm{Ta_2O_5}}\cos\varphi\right)^2} - \frac{d}{L_{\mathrm{Ta_2O_5}}\cos\varphi}\right)}{\mathrm{erfc}\left(\frac{d}{\sqrt{2}\,\sigma}\right) + \mathrm{erfc}\left(\frac{\sigma}{\sqrt{2}\,L_{\mathrm{Ta}}\cos\varphi} - \frac{d}{\sqrt{2}\,\sigma}\right) \cdot \exp\left(\frac{\sigma^2}{2(L_{\mathrm{Ta}}\cos\varphi)^2} - \frac{d}{L_{\mathrm{Ta}}\cos\varphi}\right)} \tag{S4}$$

Note that the peak region of each orbital corresponds to a different binding energy E_{BE} and corresponding kinetic energy E_{kin} of the electrons, with $E_{\mathrm{kin}} = h\nu - E_{\mathrm{BE}} - \Phi$ with photon energy $h\nu = 1486.6$ eV and work function of the electron analyzer $\Phi = 4.39$ eV. This results in a unique set of EALs $L_{\mathrm{Ta_2O_5}}(E_{\mathrm{kin}})$ and $L_{\mathrm{Ta}}(E_{\mathrm{kin}})$. Therefore, this equation is a function of the emission angle φ as well as the kinetic energy E_{kin} of the electrons. The uncertainties on the oxide thickness d and interface width σ, obtained from fitting this equation, can be minimized by increasing the number of analyzed peak regions and emission angles. A detailed error discussion and more information on this equation

can be found in reference 2. The HfO_2 layer does not need to be explicitly considered in our XPS analysis, as equation (S4) relies on relative signal intensities. Since the HfO_2 layer attenuates both the Ta^0 and Ta^{5+} signal intensities equally, its effect cancels out when calculating their ratio.

SI 4. Calculation of the effective attenuation lengths (EALs) L and number densities N

Equation (S4) (which is equal to equation (1) in the main text) requires the effective attenuation lengths (EALs) in Ta_2O_5 and Ta as an input parameter. The EAL is a measure of the attenuation of the photoelectrons inside the material, similar to the inelastic mean free path (IMFP). However, in contrast to the IMFP, the EAL also considers elastic scattering. Therefore, it is generally considered more reliable and was used in this analysis.[3] To calculate the EALs $L_{\mathrm{Ta_2O_5}}$ and L_{Ta} inside Ta_2O_5 and Ta, respectively, the universal curve proposed by Seah was used[4]

$$L = \frac{(5.8+0.0041\cdot Z^{1.7}+0.088\cdot E_{\mathrm{kin}}{}^{0.93})\ a^{1.82}}{Z^{0.38}(1-0.02\cdot E_{\mathrm{g}})} \qquad \text{(S5)}$$

Thereby, E_{kin} denotes the kinetic energy of the photoelectrons and E_{g} the band gap, both in eV. For a binary compound G_gH_h with stoichiometry coefficients g and h (e.g. for Ta_2O_5: $\mathrm{g} = 2,\ \mathrm{h} = 5$), the average atomic number Z and thickness per monolayer a are defined in the following way:[3]

$Z = \frac{\mathrm{g}Z_{\mathrm{g}}+\mathrm{h}Z_{\mathrm{h}}}{\mathrm{g}+\mathrm{h}}$ with the atomic numbers Z_{g} and Z_{h}

$a = \sqrt[3]{\frac{M}{\rho N_{\mathrm{A}}(\mathrm{g}+\mathrm{h})}}$ with the molecular weight M, mass density ρ and the Avogadro constant N_{A}.

In the case of an elemental solid, the equations simplify by $\mathrm{g} = 1$ and $\mathrm{h} = 0$.

Importantly, this equation should only be used for emission angles $\leq 65°$ and overlayer thicknesses (in our case Ta_2O_5), which reduce the substrate intensity (in our case Ta) to a maximum of 10 % of its original value. Outside of this window, the EALs can no longer be assumed constant, such that a significant error in the analysis can arise.[4]

The parameters used to calculate $L_{\mathrm{Ta_2O_5}}$ and L_{Ta} and the resulting values for all peak regions investigated can be found in the following two tables S4 and S5.

Table S4: Material parameters used to calculate L_{Ta} and $L_{\mathrm{Ta_2O_5}}$.

	$\boldsymbol{M}$ (u)	$\boldsymbol{\rho}$ (g/cm^3)	$\boldsymbol{a}$ (nm)	$\boldsymbol{E_{\mathrm{g}}}$ (eV)	$\boldsymbol{Z}$
Ta	180.95	16.7	0.26	0	73
Ta_2O_5	441.90	8.2	0.23	3.8	26.6

Table S5: Values obtained for L_{Ta} and $L_{\mathrm{Ta_2O_5}}$ for each analyzed peak region. The kinetic energy E_{kin} is related to the binding energy E_{BE} via $E_{\mathrm{kin}} = h\nu - E_{\mathrm{BE}} - \Phi$ with $h\nu = 1486.6$ eV and $\Phi = 4.39$ eV. For the binding energy, the energy of the larger peak of the doublets was used. For comparison, the IMFPs are calculated using the TPP-2M equation,[5] both for the number of valence electrons N_{V} including 4f electrons (first value, $N_{\mathrm{V,Ta}} = 19$, $N_{\mathrm{V,Ta_2O_5}} = 68$), and excluding 4f electrons (second value, $N_{\mathrm{V,Ta}} = 5$, $N_{\mathrm{V,Ta_2O_5}} = 40$).[5]

**Peak region**	$\boldsymbol{E_{\mathrm{kin,Ta}}}$ (eV)	$\boldsymbol{E_{\mathrm{kin,Ta_2O_5}}}$ (eV)	$\boldsymbol{L_{\mathrm{Ta}}}$ (nm)	$\boldsymbol{L_{\mathrm{Ta_2O_5}}}$ (nm)	$\mathbf{IMFP_{Ta}}$ (nm)	$\mathbf{IMFP_{Ta_2O_5}}$ (nm)
Ta 4f	1460.6	1455.3	1.53	1.85	1.18 – 1.84	1.72 – 1.82
Ta 5s	1412.3	1409.0	1.49	1.80	1.32 – 2.05	1.93 – 2.04
Ta 4d	1257.3	1251.5	1.35	1.63	1.44 – 2.24	2.12 – 2.23
Ta 4p	1082.8	1076.8	1.21	1.44	1.48 – 2.29	2.17 – 2.29

Additionally to the EALs, equation (S4) also requires the number densities N_{Ta^0} and $N_{\mathrm{Ta}^{5+}}$. They are defined as the number of Ta^0 or Ta^{5+} atoms per unit volume and are given in table S6.

Table S6: Values of N_{Ta^0} and $N_{\mathrm{Ta}^{5+}}$ used in the XPS analysis.

	$\boldsymbol{N}\ (\mathbf{10^{22}/cm^3})$
Ta	5.54
Ta_2O_5	2.23

SI 5. XPS peak fitting

XPS peak fitting was performed in casaXPS version 2.3.26PR1.0. Referencing was done by aligning the Fermi cutoff in the valence band to a binding energy of 0 eV.[6] Symmetric peaks were fitted using the Gaussian/Lorentzian product formula GL(*p*), for which GL(0) is a pure Gaussian and GL(100) a pure Lorentzian. All metallic peaks are asymmetric and were fitted with the Lorentzian asymmetric lineshape LA(*a,b,m*). It is defined as a convolution of a Lorentzian, with a spread to the left and right defined by the parameters *a* and *b*, and a Gaussian with width *m*.

Where possible, backgrounds were subtracted using the Analytic Shirley background. It has the advantage of showing steps at each peak, which correspond to the additional inelastic scattering of the corresponding transition, equivalently to the Shirley background. Therefore, the background is also physically justified for regions with several peaks. To take account of broad underlying peaks in the background, the Analytic Shirley background has four cross-section parameters. Three of them define the linear, quadratic and cubic coefficients of a cubic polynomial with which the step height is modified. The fourth parameter is an offset in energy. However, the Analytic Shirley background always ends horizontally toward the lower binding energy side. For regions lying on a tilted background, this leads to errors which can significantly change the intensity ratios. Therefore, the backgrounds of the Ta 5s and O 1s regions, which require a tilted background and only span a small energy range, were subtracted using an Offset Shirley background.[7] In the case of the Ta 4p region, the peak region is large and lies on top of a very pronounced peak. Therefore, a Spline Tougaard background was used, which allows for the most flexibility to adapt the shape of the background.[7]

For spin-orbit split peak doublets, the ratio of the peak intensities was fixed according to their degeneracy, given by 2J+1: For the 4f-orbitals the intensity ratio is given by $I(4f_{7/2})/I(4f_{5/2}) = 4/3$, for 4d-orbitals it is $I(4d_{5/2})/I(4d_{3/2}) = 3/2$ and for 4p-orbitals it is $I(4p_{3/2})/I(4p_{1/2}) = 2/1$.

In the Ta 4f peak region, O^{2-} 2s (at 22.3 eV in the 2 nm HfO_2 samples) and In^{3+} 4d (at 18.9 and 19.7 eV in the 2 nm HfO_2 samples) peaks are buried below the other peaks. To minimize errors on the Ta^0 4f and Ta^{5+} 4f peak intensities, the intensities of the O^{2-} 2s and In^{3+} 4d peaks were estimated in the following way: First, the areas of the O^{2-} 1s and In^{3+} 3d peaks were determined in the O 1s and Ta 4p peak region, respectively, where these peaks can be fitted reliably (see tables S6 and S7*)*. Then, the corresponding areas of the O^{2-} 2s and In^{3+} 4d peaks were calculated using the corrected relative sensitivity factor (RSF) values at 55 eV pass energy for our instrument. The resulting areas were then fixed in the Ta 4f peak region. For simplicity, the O^{2-} 2s peak was thereby only fitted with one asymmetric peak shape instead of the two symmetric peaks corresponding to the carbonate and hydroxide subpeaks.

Tables S7 and S8 provide an overview of all fit parameters of the samples with 2 and 3 nm HfO_2, respectively. The binding energies and the full width at half maximum (FWHM) values were averaged over all emission angles and gate voltages and the average and standard deviation are given in the tables.

Table S7. For sample Si/ SiO_2/ Ta (15)/ HfO_2 (2): Binding energies, line shapes, full width at half maximum (FWHM) and background types for the orbitals in all analyzed peak regions. For the binding energy and FWHM, the average and standard deviation of the values from all angles and all gate voltages are provided (if smaller than 0.1 eV, 0.1 eV is estimated as the uncertainty). Large uncertainties in the FWHM are typically caused by large differences in peak size after different gating steps (the smaller the peak, the smaller its FWHM).

Peak region	Orbital	Binding energy (eV)	Line shape	FWHM (eV)	Background type
Ta 4f	Hf^{4+} $4f_{7/2}$	17.8 ± 0.1	GL(40)	1.2 ± 0.1	Analytic Shirley
	Hf^{4+} $4f_{5/2}$	19.5 ± 0.2	GL(40)	1.2 ± 0.1	
	Ta^0 $4f_{7/2}$	21.6 ± 0.1	LA(1,7,50)	0.8 ± 0.1	
	Ta^0 $4f_{5/2}$	23.5 ± 0.1	LA(1,7,50)	0.8 ± 0.1	
	Ta^{5+} $4f_{7/2}$	26.9 ± 0.2	GL(40)	1.3 ± 0.2	
	Ta^{5+} $4f_{5/2}$	28.8 ± 0.1	GL(40)	1.3 ± 0.2	
	O^{2-} 2s	22.3 ± 0.2	LA(1,7,150)	5.5 ± 0.1	
	In^{3+} $4d_{5/2}$	18.9 ± 0.2	GL(50)	0.7 ± 0.1	
	In^{3+} $4d_{3/2}$	19.7 ± 0.2	GL(50)	0.7 ± 0.1	
O 1s	O^{2-} 1s	531.1 ± 0.2	GL(40)	1.4 ± 0.1	Offset Shirley
	O 1s (O-C)	532.6 ± 0.2	GL(40)	2.0 ± 0.1	
Ta 5s	Hf^{4+} 5s	65.9 ± 0.2	GL(75)	4.6 ± 0.9	Offset Shirley
	Ta^0 5s	69.9 ± 0.2	LA(1.4,6,80)	4.2 ± 1.2	
	Ta^{5+} 5s	73.2 ± 0.2	GL(95)	3.9 ± 1.0	
Ta 4d	Hf^{4+} $4d_{5/2}$	214.0 ± 0.2	GL(80)	4.1 ± 0.1	Analytic Shirley
	Hf^{4+} $4d_{3/2}$	224.9 ± 0.2	GL(80)	4.6 ± 0.3	
	Ta^0 $4d_{5/2}$	224.9 ± 0.2	LA(1.3,2,50)	3.6 ± 0.9	
	Ta^0 $4d_{3/2}$	238.0 ± 0.2	LA(1.3,2,50)	4.0 ± 0.8	
	Ta^{5+} $4d_{5/2}$	230.7 ± 0.3	GL(95)	3.8 ± 0.6	
	Ta^{5+} $4d_{3/2}$	242.5 ± 0.1	GL(80)	3.8 ± 0.8	
Ta 4p	Hf^{4+} $4p_{3/2}$	382.7 ± 0.1	GL(85)	5.0 ± 0.1	Spline Tougaard
	Hf^{4+} $4p_{1/2}$	439.9 ± 0.1	GL(95)	7.3 ± 0.2	
	Hf^{4+} (sat)	396.4 ± 0.2	GL(30)	5.8 ± 0.7	
	Hf^{4+} (sat)	453.1 ± 0.5	GL(30)	6.0 ± 0.7	
	Ta^0 $4p_{3/2}$	399.4 ± 0.2	LA(1,7,150)	4.7 ± 1.2	
	Ta^0 $4p_{1/2}$	458.9 ± 1.1	LA(1,7,150)	7.7 ± 2.6	
	Ta^0 (sat)	421.1 ± 0.7	GL(30)	9.4 ± 1.6	
	Ta^0 (sat)	484.6 ± 1.7	GL(30)	8.7 ± 2.8	
	Ta^{5+} $4p_{3/2}$	405.4 ± 0.3	GL(77)	6.0 ± 1.1	
	Ta^{5+} $4p_{1/2}$	466.4 ± 0.4	GL(77)	8.8 ± 1.9	
	In^{3+} $3d_{5/2}$	445.9 ± 0.1	GL(50)	1.5 ± 0.1	
	In^{3+} $3d_{3/2}$	453.4 ± 0.2	GL(50)	1.5 ± 0.1	

Table S8. For sample Si/ SiO_2/ Ta (15)/ HfO_2 (3): Binding energies, line shapes, FWHM and background types for the orbitals in all analyzed peak regions, equivalent to Table S7 for the sample with 2 nm HfO_2.

Peak region	Orbital	Binding energy (eV)	Line shape	FWHM (eV)	Background type
Ta 4f	Hf^{4+} $4f_{7/2}$	18.1 ± 0.1	GL(30)	1.2 ± 0.1	Analytic Shirley
	Hf^{4+} $4f_{5/2}$	19.8 ± 0.1	GL(40)	1.2 ± 0.2	
	Ta^0 $4f_{7/2}$	21.7 ± 0.2	LA(1,7,50)	0.7 ± 0.1	
	Ta^0 $4f_{5/2}$	23.6 ± 0.2	LA(1,7,50)	0.7 ± 0.1	
	Ta^{5+} $4f_{7/2}$	27.2 ± 0.1	GL(40)	1.9 ± 0.7	
	Ta^{5+} $4f_{5/2}$	29.0 ± 0.1	GL(40)	1.9 ± 0.7	
	O^{2-} 2s	22.1 ± 0.1	LA(1,7,150)	5.4 ± 0.3	
	In^{3+} $4d_{5/2}$	19.8 ± 0.1	GL(50)	1.0 ± 0.1	
	In^{3+} $4d_{3/2}$	20.6 ± 0.1	GL(50)	1.0 ± 0.1	
O 1s	O^{2-} 1s	531.4 ± 0.1	GL(40)	1.4 ± 0.1	Offset Shirley
	O 1s (O-C)	532.9 ± 0.1	GL(40)	2.0 ± 0.1	
Ta 5s	Hf^{4+} 5s	66.3 ± 0.2	GL(75)	4.8 ± 0.6	Offset Shirley
	Ta^0 5s	70.3 ± 0.1	LA(1.4,6,80)	3.2 ± 0.9	
	Ta^{5+} 5s	73.2 ± 0.2	GL(95)	2.8 ± 1.4	
Ta 4d	Hf^{4+} $4d_{5/2}$	214.4 ± 0.1	GL(80)	4.2 ± 0.1	Analytic Shirley
	Hf^{4+} $4d_{3/2}$	225.0 ± 0.1	GL(80)	4.9 ± 0.4	
	Ta^0 $4d_{5/2}$	225.4 ± 0.2	LA(1.3,2,50)	3.0 ± 0.5	
	Ta^0 $4d_{3/2}$	238.1 ± 0.2	LA(1.3,2,50)	4.1 ± 0.7	
	Ta^{5+} $4d_{5/2}$	230.9 ± 0.3	GL(95)	4.9 ± 0.6	
	Ta^{5+} $4d_{3/2}$	242.7 ± 0.3	GL(85)	3.3 ± 1.2	
Ta 4p	Hf^{4+} $4p_{3/2}$	383.0 ± 0.1	GL(85)	4.9 ± 0.1	Spline Tougaard
	Hf^{4+} $4p_{1/2}$	440.3 ± 0.1	GL(95)	7.5 ± 0.2	
	Hf^{4+} (sat)	396.7 ± 0.1	GL(30)	6.0 ± 0.8	
	Hf^{4+} (sat)	454.2 ± 0.4	GL(30)	5.5 ± 0.8	
	Ta^0 $4p_{3/2}$	399.6 ± 0.3	LA(1,7,150)	5.1 ± 1.0	
	Ta^0 $4p_{1/2}$	459.6 ± 0.6	LA(1,7,95)	7.6 ± 2.5	
	Ta^0 (sat)	420.7 ± 0.2	GL(30)	7.6 ± 1.4	
	Ta^0 (sat)	487.1 ± 0.9	GL(30)	3.6 ± 1.6	
	Ta^{5+} $4p_{3/2}$	405.6 ± 0.3	GL(77)	7.1 ± 1.2	
	Ta^{5+} $4p_{1/2}$	465.3 ± 0.8	GL(77)	7.9 ± 2.7	
	In^{3+} $3d_{5/2}$	446.2 ± 0.1	GL(50)	1.4 ± 0.1	
	In^{3+} $3d_{3/2}$	453.7 ± 0.1	GL(50)	1.4 ± 0.1	

SI 6. XPS survey spectra showing the full measured energy range

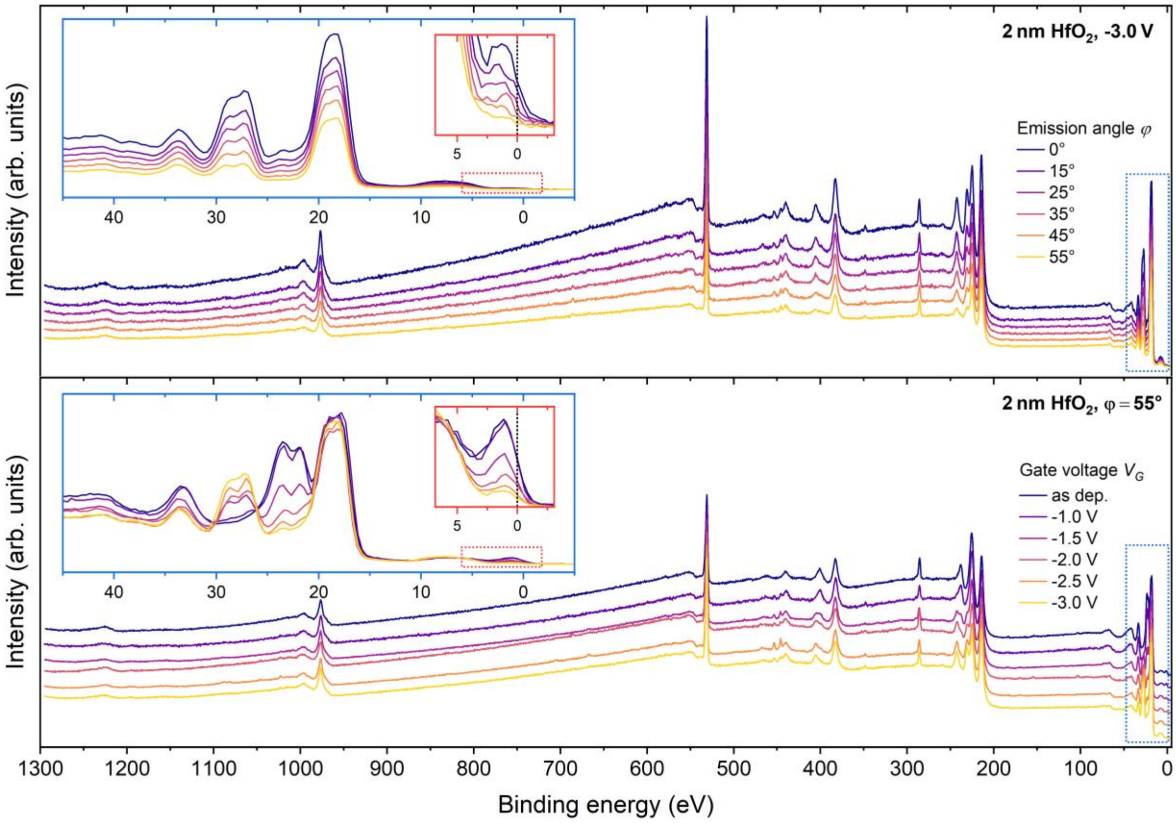

Figure S2: Survey spectra of the samples with 2 nm HfO_2. Referencing of the binding energy scale was performed by aligning the Fermi cutoff in the valence band to a binding energy of 0 eV, as shown in the inset framed in red. The spectra in the bottom panel (2 nm HfO_2, $\varphi = 55°$) were normalized and shifted vertically on the intensity scale for clarity.

SI 7. Overview over the XPS fits for all peak regions at all angles and gate voltages

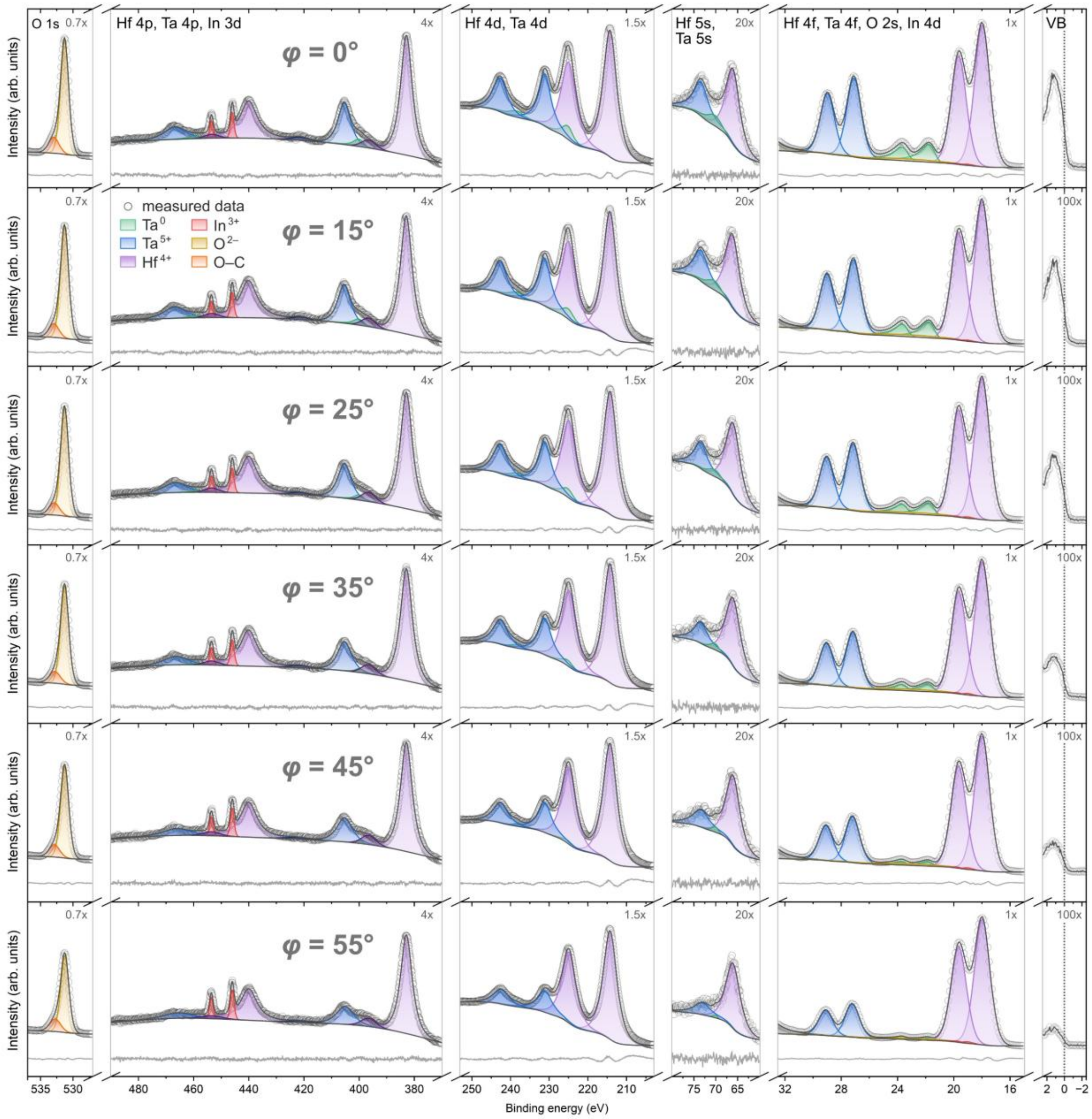

Figure S3: Overview of the XPS spectra for the sample with 2 nm HfO_2 after -3 V gating for all analyzed peak regions at all angles.

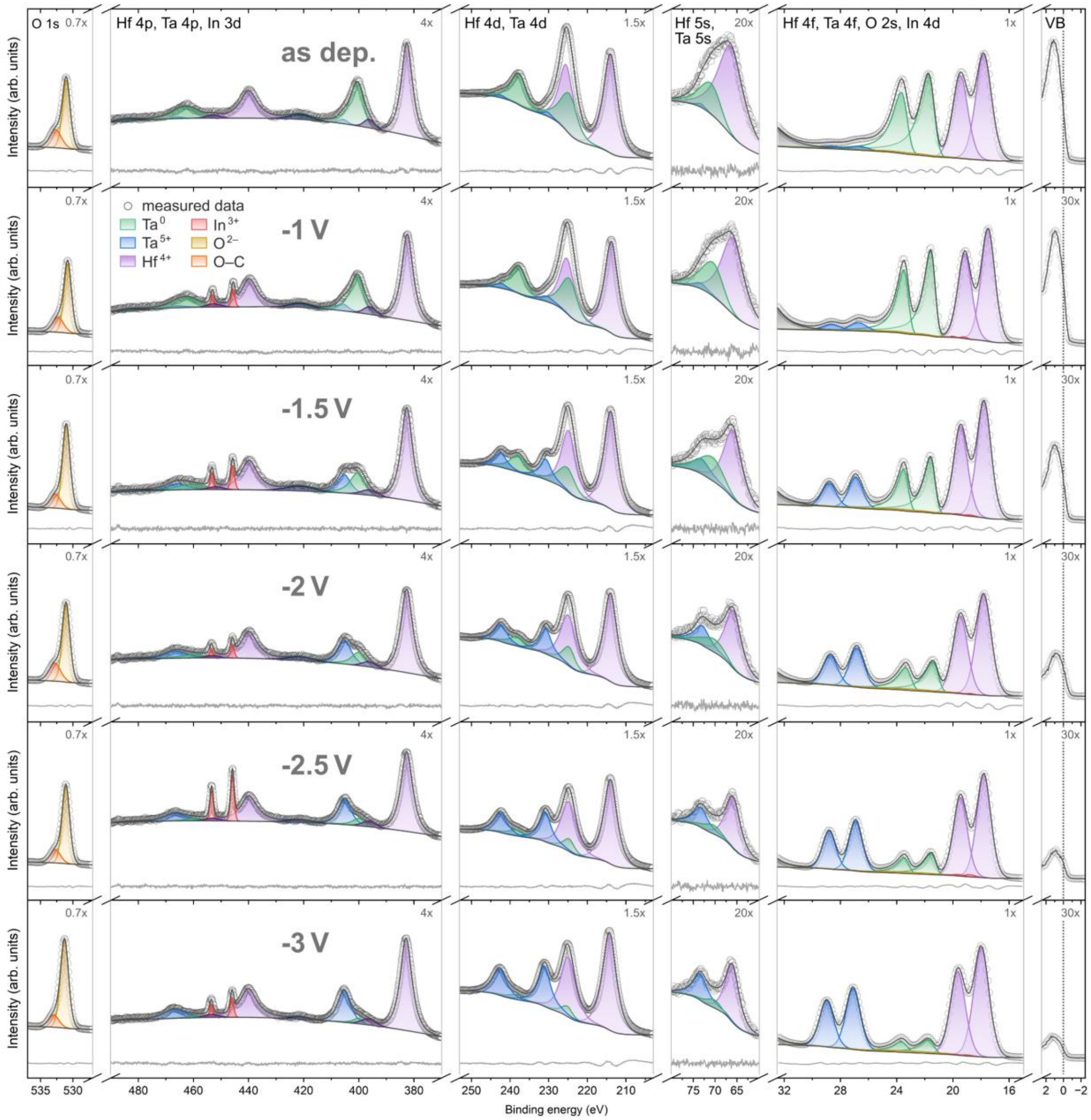

Figure S4: Overview of the XPS spectra for the sample with 2 nm HfO_2 at 0° emission angle for all analyzed peak regions at all gating steps.

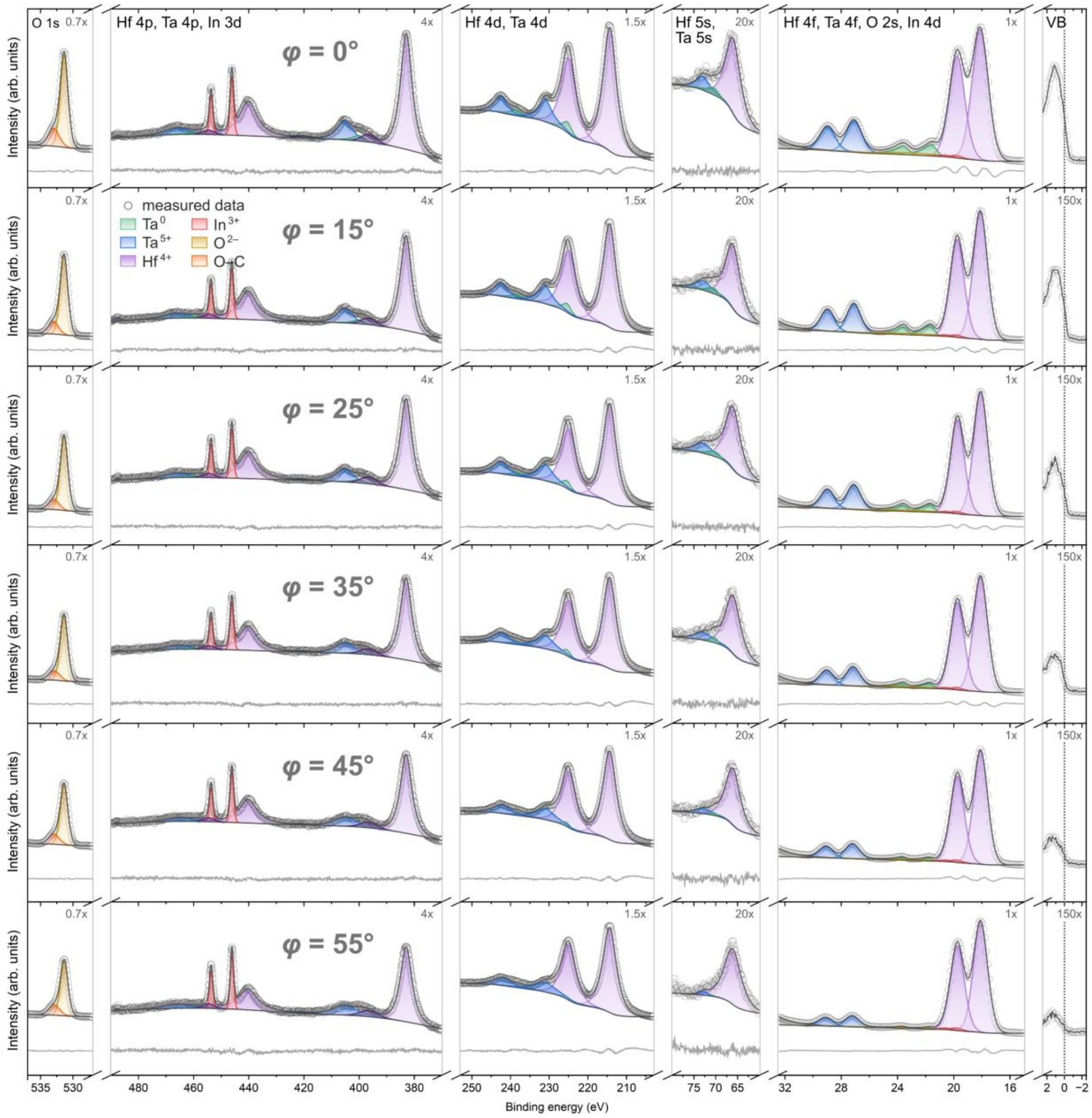

Figure S5: Overview of the XPS spectra for the sample with 3 nm HfO_2 after -3 V gating for all analyzed peak regions at all angles.

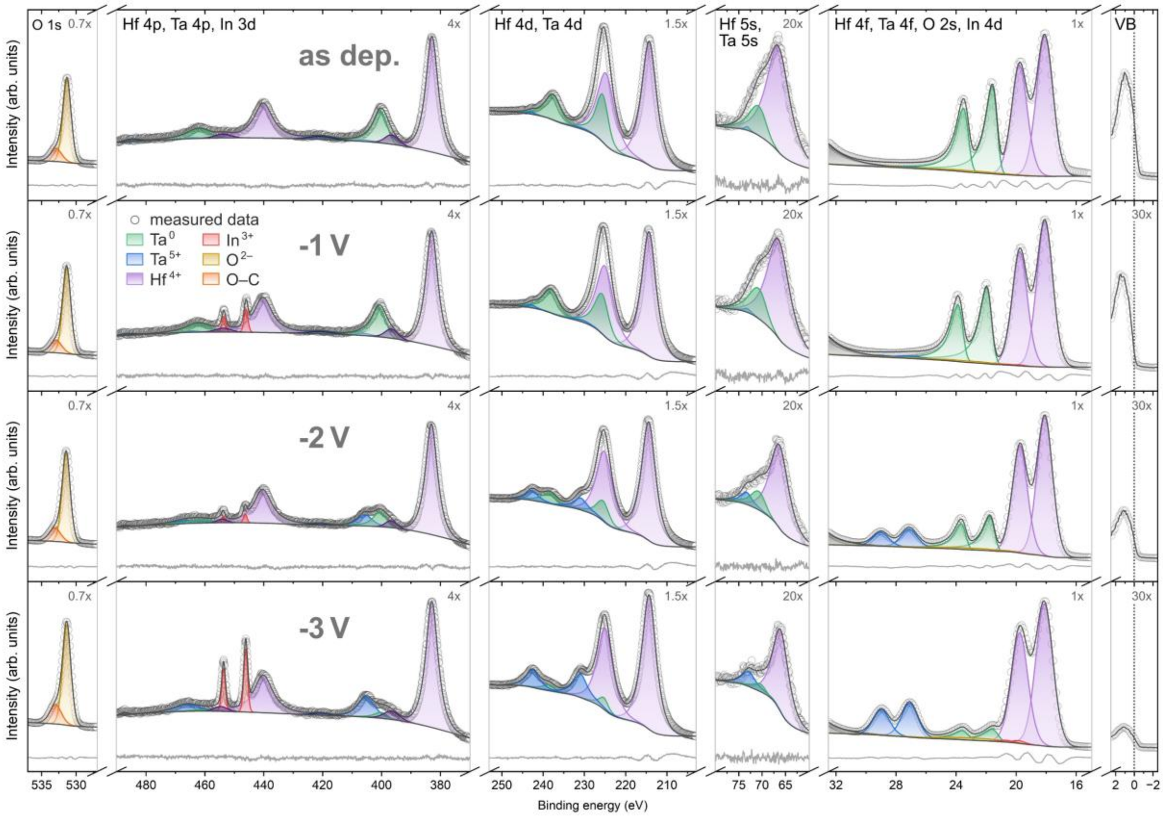

Figure S6: Overview of the XPS spectra for the sample with 3 nm HfO_2 at 0° emission angle for all analyzed peak regions at all gating steps.

SI 8. Calculation of the scattering length density (SLD)

In Figure 4a of the manuscript, we show an SLD profile calculated from the XPS results for a direct comparison to the SLD obtained from XRR experiments. The SLD, as given in XRR, is the measure of how strongly a material scatters incoming X-rays. It is given by the electron density ρ_e multiplied by the electron radius r_e. To calculate the electron density, the scattering factors f_0 (atomic form factor, approximately equal to the atomic number Z for small angles θ toward the sample surface) and f' (dispersion correction) have to be considered for each element. The sum of the two $f_1(E) = (f_0 + f')(E)$ as a function of the energy of the incoming X-rays is tabulated on the website 'The atomic scattering factors' provided by the Berkeley lab.[8] The first column gives the energy in eV, the second column gives the scattering factors $f_1 = (f_0 + f')$ and the third column gives the anomalous dispersion factor f'' which takes absorption into account and is not required here.

The electron density can be calculated from the product of the scattering factors $(f_0 + f')$ and the number density N of the respective element. For compounds with several elements, the SLD is a function of the energy E and is therefore given as

$$\mathrm{SLD}(E) = r_\mathrm{e}\, N_\mathrm{FU} \sum_{i\in\mathrm{FU}} (f_0 + f')_i(E) \cdot m_i \tag{S6}$$

with the number density N_FU of the formula unit and the stoichiometry coefficients m_i of each element in the formula unit.

To calculate the SLD from the XPS depth profiles, the number densities N_{Ta^0} and $N_{\mathrm{Ta}^{5+}}$ obtained from XPS as a function of the z-position were used (see table S6). The number density of Ta_2O_5 is obtained from $N_{\mathrm{Ta_2O_5}} = N_{\mathrm{Ta}^{5+}}/2$. Table S9 provides the parameters for the calculation of the SLDs of pure Ta and Ta_2O_5. The SLD profile at the Ta_2O_5/Ta interface is obtained by including the z-dependence of the number densities and including both Ta and Ta_2O_5 in the sum defined by equation (S6).

Table S9: Parameters for the calculation of the SLDs of pure Ta and Ta_2O_5 at the energy of Cu Kα radiation (8.05 keV) as used in XRR. The values of $(f_0 + f')_i$ were obtained from the table from the Berkeley lab at 8047.42 eV for Ta and 8048.79 eV for O.

	N_FU $(10^{22}/\mathrm{cm}^3)$	m_Ta	$(f_0 + f')_\mathrm{Ta}$	m_O	$(f_0 + f')_\mathrm{O}$	**SLD** $(r_e/\mathrm{Å}^3)$
Ta	5.54	1	67.51	0	8.05	3.74
Ta_2O_5	1.12	2	67.51	5	8.05	1.96

SI 9. Investigation of intermixing between Hf and Ta

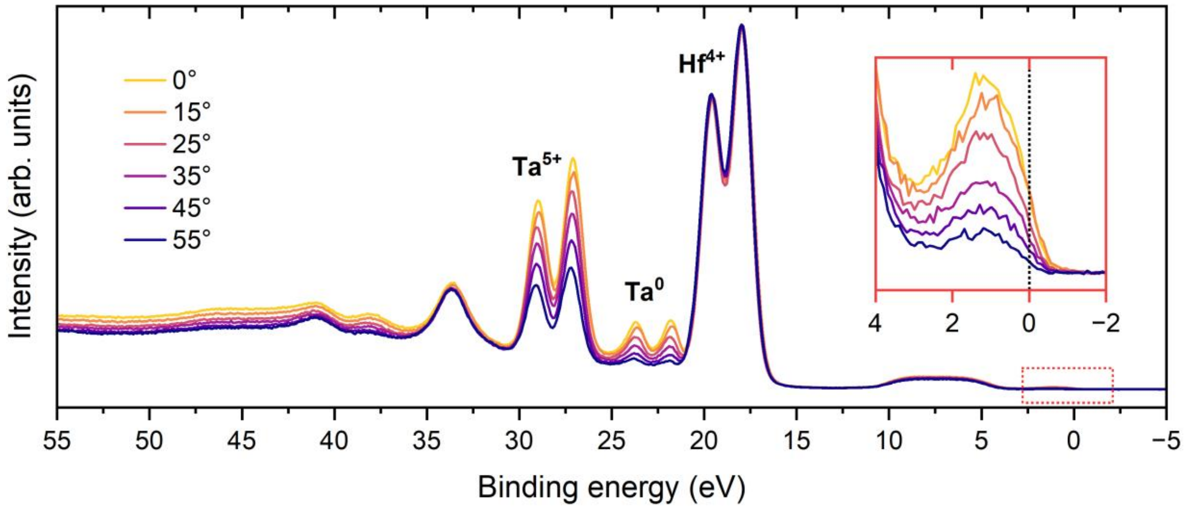

Figure S7: Comparison between the Ta 4f and Hf 4f XPS peaks at all angles for the sample with 2 nm HfO_2. The data was normalized to the Hf^{4+} peaks. Like the Ta^0 peaks, the Ta^{5+} peaks show a clear reduction of the signal for increasing incidence angles. This reveals that the Ta^{5+} signal comes from a lower thickness compared to the Hf^{4+} signal. This leads to the conclusion that the Ta_2O_5 layer forms at the interface between Ta and HfO_2 and there is no significant penetration of Ta cations into the HfO_2 layer. The inset shows the Fermi edge, which was used for referencing of the binding energy scale.

REFERENCES

(1) Glavic, A.; Björck, M. GenX 3: The Latest Generation of an Established Tool. *J. Appl. Crystallogr.* **2022**, *55* (4), 1063–1071. https://doi.org/10.1107/S1600576722006653.

(2) Wortmann, M.; Bednarz, B.; Nezafat, N. B.; Viertel, K.; Kuschel, O.; Schmalhorst, J.; Ennen, I.; Gärner, M.; Frese, N.; Jakob, G.; Wollschläger, J.; Schierning, G.; Hütten, A.; Kuschel, T. Oxidation State Depth Profiling by Self-Consistent Fitting of All Emission Peaks in the X-Ray Photoelectron Spectrum of SnTe. *Appl. Surf. Sci.* **2025**, *713*, 164356. https://doi.org/10.1016/j.apsusc.2025.164356.

(3) Jablonski, A.; Powell, C. J. Effective Attenuation Lengths for Different Quantitative Applications of X-Ray Photoelectron Spectroscopy. *J. Phys. Chem. Ref. Data* **2020**, *49* (3), 033102. https://doi.org/10.1063/5.0008576.

(4) Seah, M. P. Simple Universal Curve for the Energy-Dependent Electron Attenuation Length for All Materials. *Surf. Interface Anal.* **2012**, *44* (10), 1353–1359. https://doi.org/10.1002/sia.5033.

(5) Tanuma, S.; Powell, C. J.; Penn, D. R. Calculation of Electron Inelastic Mean Free Paths (IMFPs) VII. Reliability of the TPP-2M IMFP Predictive Equation. *Surf. Interface Anal.* **2003**, *35* (3), 268–275. https://doi.org/10.1002/sia.1526.

(6) Greczynski, G.; Hultman, L. Binding Energy Referencing in X-Ray Photoelectron Spectroscopy. *Nat. Rev. Mater.* **2025**, *10* (1), 62–78. https://doi.org/10.1038/s41578-024-00743-5.

(7) CasaXPS. Peak Fitting in XPS. http://www.casaxps.com/help_manual/manual_updates/peak_fitting_in_xps.pdf (accessed 2025-08-19).

(8) Berkeley Lab. The Atomic Scattering Factors. https://henke.lbl.gov/optical_constants/asf.html (accessed 2025-07-30).
